# On the usefulness of mock genomes to define heterotic pools, testers, and hybrid predictions in orphan crops

**DOI:** 10.3389/fpls.2023.1164555

**Published:** 2023-06-02

**Authors:** Ingrid Pinheiro Machado, Júlio César DoVale, Felipe Sabadin, Roberto Fritsche-Neto

**Affiliations:** ^1^ Department of Crop Science, Federal University of Ceará, Fortaleza, Brazil; ^2^ School of Plant and Environmental Sciences, Virginia Tech: Virginia Polytechnic Institute and State University, Blacksburg, VA, United States; ^3^ LSU AgCenter, Louisiana State University Agricultural Center, Baton Rouge, LA, United States

**Keywords:** genotyping-by-sequencing, SNP-array, formation of heterotic groups, genomic prediction of single-crosses, minor crops, underused crops, simulated genome

## Abstract

The advances in genomics in recent years have increased the accuracy and efficiency of breeding programs for many crops. Nevertheless, the adoption of genomic enhancement for several other crops essential in developing countries is still limited, especially for those that do not have a reference genome. These crops are more often called orphans. This is the first report to show how the results provided by different platforms, including the use of a simulated genome, called the mock genome, can generate in population structure and genetic diversity studies, especially when the intention is to use this information to support the formation of heterotic groups, choice of testers, and genomic prediction of single crosses. For that, we used a method to assemble a reference genome to perform the single-nucleotide polymorphism (SNP) calling without needing an external genome. Thus, we compared the analysis results using the mock genome with the standard approaches (array and genotyping-by-sequencing (GBS)). The results showed that the GBS-Mock presented similar results to the standard methods of genetic diversity studies, division of heterotic groups, the definition of testers, and genomic prediction. These results showed that a mock genome constructed from the population’s intrinsic polymorphisms to perform the SNP calling is an effective alternative for conducting genomic studies of this nature in orphan crops, especially those that do not have a reference genome.

## 1 Introduction

Molecular markers have been used to develop genomic tools to improve economically important crops (Mammadov et al., 2012; Thomson, 2014). Currently, single-nucleotide polymorphism (SNP) markers are the most used in genomic studies (Fritsche-Neto et al., 2021), as they provide higher resolution due to their frequent occurrence and uniformity throughout the genome (Gupta et al., 2008). Rapid advances in next-generation sequencing (NGS) technologies, combined with high levels of diversity in SNP, have made it possible to develop high-throughput genotyping platforms (Bachlava et al., 2012).

There are several genotyping platforms for obtaining SNPs throughout the genome, which have provided an infinity of sequencing information with remarkable improvements in coverage, time, and costs, making it possible to genotype thousands of samples with many markers (Bevan and Uauy, 2013), with SNP array and NGS platforms being the most appropriate for this purpose (Rasheed et al., 2017). There are many array-based genotyping platforms available in major crops such as maize (Unterseer et al., 2014), wheat (Winfield et al., 2016), rice (Singh et al., 2015), and soybean (Lee et al., 2015). These platforms have many advantages, such as fast scans with high call rates and density. However, they present an investigation bias when the set of individuals does not faithfully represent the genetic diversity explored in the study panel. Furthermore, it has a high cost and is inaccessible to small breeding programs (Messing and Dooner, 2006; Frascaroli et al., 2013), especially those of unprofitable crops.

Beyond crop-specific SNP arrays, NGS-based platforms are adaptable to various crops, regardless of prior knowledge of genomics, genome size, organization, or ploidy (Rasheed et al., 2017). Genotyping-by-sequencing (GBS) appears as an alternative to overcome the verification bias since it is based on sequencing and, therefore, allows the discovery of alleles in the diversity panel analyzed, in addition to having a lower cost when compared to SNP array. However, GBS generates many low-quality markers with a high rate of lost data (Heslot et al., 2013). The advances in genomics in recent years have increased the accuracy and efficiency of breeding programs for many crops. However, adopting genomic enhancement for several other staple crops essential in developing countries is still limited, especially for traits under complex genetic control, which are crucial to crop performance (Varshney et al., 2012). This is because most studies use the array-based and GBS-based SNP marker approach, which depends on a reference genome for SNP discovery. Crops that do not have a reference genome cannot take advantage of biotechnological tools to improve their genetic gain and develop modern cultivars faster (Armstead et al., 2009).

There are many crops of unique relevance to developing countries, essential for the food, nutritional, and economic security of these countries, which still do not have a reference genome (Baldermann et al., 2016; Hendre et al., 2019). These crops are more often called orphans. The term “orphan” is derived from the condition of neglect and helplessness of these crops by the scientific community, leading to the designation of such species as underused, neglected, or minor crops (Tadele and Assefa, 2012). GBS also appears as an option for genomic studies in these crops, especially when they do not have a reference genome (Sabadin et al., 2022). With these data, it is possible to build a mock reference to perform the SNP calling, where the discovery of polymorphisms will be intrinsic to the study population without using an external genome (Melo et al., 2016). This pipeline has already been successfully used in several genomic studies (Adhikari et al., 2018; Holloway et al., 2018; Munjal et al., 2018; Matias et al., 2019; Sabadin et al., 2022). Adopting this technology in poorly studied crops has a tangible impact on the progress of the breeding process (Ye and Fan, 2021).

Recent studies have compared the performance of genotyping platforms and how this choice affects genomic studies regarding genetic diversity studies (Elbasyoni et al., 2018; Darrier et al., 2019), genome-wide association study (GWAS) (Negro et al., 2019), and genomic prediction (Chu et al., 2020). Only one report compares the performance of the mock reference pipeline with standard genotyping approaches in genomic prediction studies (Sabadin et al., 2022). However, this study used a relatively small germplasm panel. It did not consider the effect of genotyping platforms on population structure, the formation of heterotic groups, and the choice of testers, which is fundamental for a breeding program that wants to synthesize single crosses.

Although some reports are available, there still needs to be a consensus on how different platforms provide the results. Thus, we conducted a full study on this topic with a robust germplasm panel. For this, we compared different genotyping scenarios from the beginning of the breeding process with the approach of genetic diversity and population structure, advancing to the formation of heterotic groups and choice of testers to the synthesis and prediction of single crosses. Thus, this information will be valuable to leverage genomic studies and accelerate the development of cultivars in minor crops without a reference genome. Therefore, our goals were to verify whether the source of SNP can influence the assessment of the population structure of parental lines, to ascertain if the source of SNP can affect the determination of heterotic groups and the prediction of single crosses performance, and check if the GBS and the mock genome efficiently performs the SNP calling in orphan crops (without reference genome).

## 2 Materials and methods

**Figure 1:**
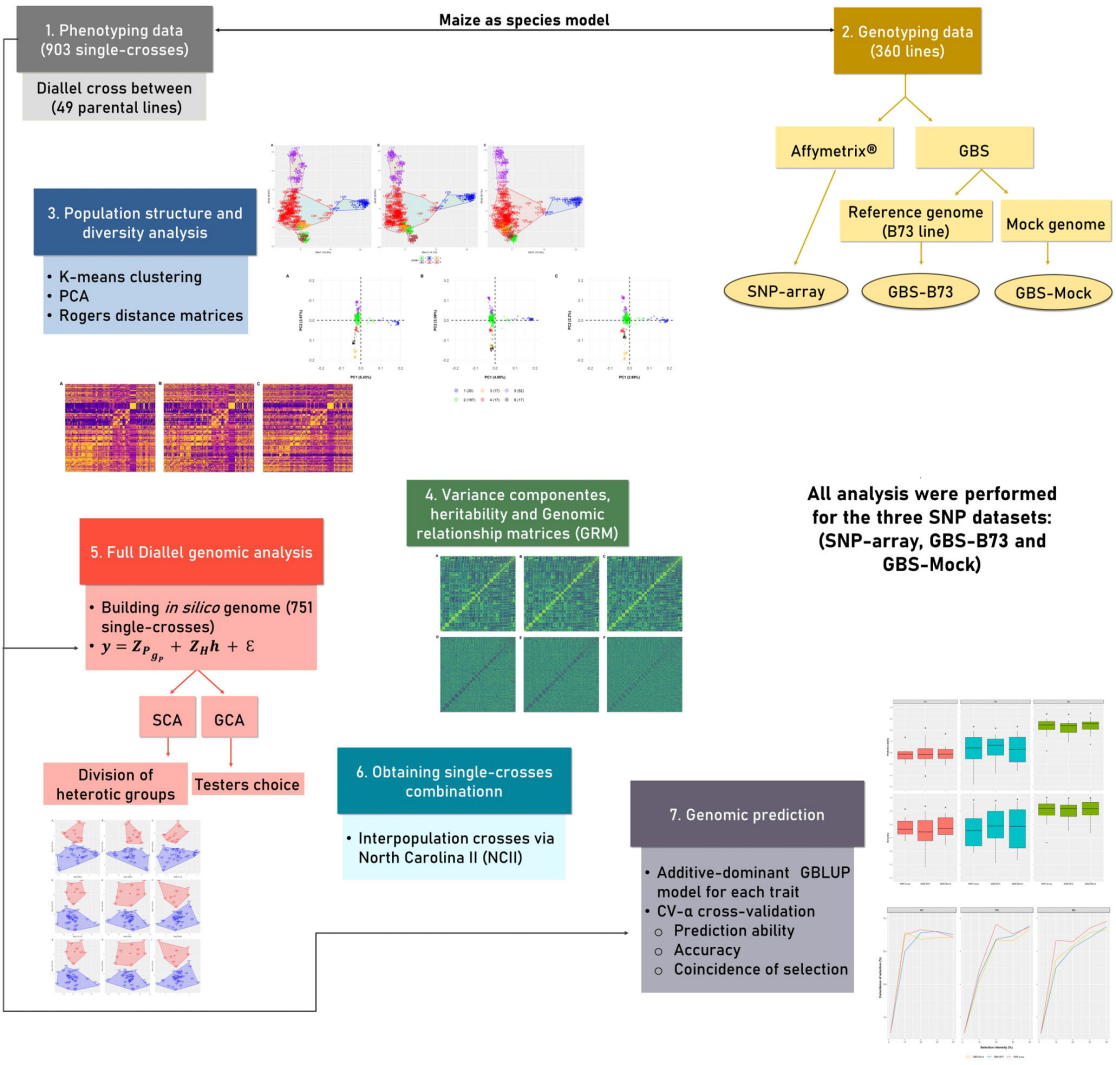
The workflow of analysis performed in the study. The different colors represent different stages of the analysis. Genomic information from the three SNP datasets was used in all analyses. SNP, single-nucleotide polymorphism.

To facilitate the understanding of the analysis carried out in this study, a workflow is described in **Figure 1**. Each stage of the analysis is detailed in the following sections.

### 2.1 Species model

We used maize as a model species in this study because it is already well-established regarding SNP array, with several array options available (Ganal et al., 2011; Unterseer et al., 2014; Xu et al., 2017), and GBS protocols are also well-established for this species (Crossa et al., 2013; Li et al., 2015; Wang et al., 2020). In addition, maize has a diverse, complex, and dynamic genome (Schnable et al., 2009), which is suitable for this study. We used a public panel of tropical maize germplasm containing 360 parental lines (Yassue et al., 2021a). The genomic and phenotypic information about this panel is available on the Mendeley platform (https://data.mendeley.com/datasets/5rtc89t7v5/1).

### 2.2 Phenotypic data

The phenotypic dataset consists of 903 maize single crosses (Fritsche-Neto et al., 2019) derived from a diallel cross between 49 parental lines to a public tropical maize diversity, selected based on nitrogen use efficiency (Mendonça et al., 2017). Field trials were carried out in Anhembi (22°50′51″S, 48°01′06″W) and Piracicaba (22°42′23″S, 47°38′12″W), in the State of São Paulo, during the second growing season, from January to June 2016 and 2017. Single crosses were evaluated in an augmented block design, where each block consisted of 16 single crosses and two checks (commercial single crosses). In both locations and years, the single crosses were evaluated under two nitrogen (N) conditions, low N with 30 kg N ha^−1^ and ideal N with 100 kg N ha^−1^. Each location × year × N level combination was defined as an environment.

Each plot consisted of 7 m rows spaced 0.50 m apart. Conventional fertilization and weed and pest control were carried out. The traits evaluated were grain yield (GY, mg ha^−1^), plant height (PH, cm), and ear height (EH, cm). The plots were harvested manually, and the grains were harvested with a moisture content of approximately 18%. Subsequently, grain yield was corrected for 13% moisture, according to Mulvaney and Devkota (2020). More details on the phenotypic dataset’s experimental design and cultivation practices were previously reported by Fritsche-Neto et al. (2018) and Galli et al. (2020).

### 2.3 Genetic-statistical model for obtaining BLUEs

The joint analysis of each trait was performed to estimate the means of the single crosses across the environments. Thus, an equation was adjusted to obtain the Best Linear Unbiased Estimator (BLUE) for each genotype, and later, the adjusted means of these across the environments evaluated by the following mixed model were estimated:


y=Ql+Sb+Tc+Ug+Vi+ϵ,

where ***y*** is the vector of phenotypic values of single crosses and checks; ***l*** is the vector of fixed effects of the environment (site × year × N level combination); *b* is the vector of random effects of block nested within environments, where *b* ~ *N*(0, *I*σ^2^_b_); *c* is the vector of fixed effects of checks; *g* is the vector of fixed effects of single crosses; *i* is the vector of fixed effects of the interaction checks × environments; *ε* is the vector of random residual effects, where *ϵ* ~ *N*(*0*, *De*). An unstructured covariance matrix across environments was assumed for the residual term (*De*) due to the contrasting doses of N. *Q*, *S*, *T*, *U*, and *V* are the incidence matrices for *l*, *b*, *c*, *g*, and *i*. The analysis was performed using the *ASReml-R* (Butler et al., 2018).

### 2.4 Genotypic data and analysis

The 360 lines belonging to the public tropical maize diversity mentioned above were genotyped using two SNP high-density genotyping platforms: 1) Affymetrix^®^ Axiom Maize Genotyping Array (SNP array) and 2) GBS method following the protocol described by Poland et al. (2012). In this last method, individual samples of genomic DNA were digested by two restriction enzymes, *Pst*I and *Mse*I, to reduce the genome complexity uniformly. Subsequently, the samples were included in a sequencing plate, performed on the Illumina NextSeq 500 platform (Illumina Inc., San Diego, CA, USA).

The raw GBS data were used for two purposes: the first was to perform the SNP calling using the B73 line of temperate germplasm as a reference genome. The second purpose was to build a simulated reference genome (mock genome) according to the GBS-SNP-CROP pipeline proposed by Melo et al. (2016) and use it to perform the SNP calling. This pipeline aggregates custom analysis and filtering procedures with bioinformatics tools on raw GBS readings. The method employs a variant calling strategy based on patterns of polymorphisms within the individual or cluster and across populations or clusters to identify sequencing or PCR errors. Finally, the pipeline uses a reading grouping strategy based on similarity to generate representative sequences, that is, a simulated reference of GBS fragments. Details of each stage of mock genome building can be found in Melo et al. (2016).

Further analysis was performed considering three SNP datasets: 1) SNP array, 2) GBS with SNP call using B73 as the reference genome (GBS-B73), and 3) GBS with the simulated genome being used as the reference genome (GBS-Mock). For GBS datasets, according to standard parameters, SNPs were scored from raw data using the TASSEL 5.0 GBSv2 pipeline (Glaubitz et al., 2014). With the use of the Burrows-Wheeler Alignment tool (BWA) (Li and Durbin, 2009), the tags were aligned against the reference genome (GBS-B73 and GBS-Mock).

As two genotyping platforms (SNP array and GBS) were performed, the parental lines that showed a very contrasting genotypic profile between the two platforms were removed from the analysis to obtain a fair comparison. Thus, between sequencing errors and divergences in genotypic profiles between platforms, 330 parental lines remained, among which 45 parental lines make up the diallel, which generated 751 single crosses. The number of markers concerning the raw data was 18,413 (SNP array), 131,350 (GBS-B73), and 46,9126 (GBS-Mock). All SNP sets underwent quality control, in which low call rates (<90%) and non-biallelic markers were removed from the datasets. The remaining missing data were imputed by the Beagle 5.0 algorithm (Browning et al., 2018). Pairwise linkage disequilibrium was calculated as the correlation of allele frequencies squared (r^2^), and values greater than 0.99 were removed from the datasets using the *SNPRelate* package (Zheng et al., 2012), resulting in 12,704 (SNP array), 11,153 (GBS-B73, and 4,935 (GBS-Mock) SNP markers.

Subsequently, new quality control was performed, in which heterozygous loci in at least one individual were removed. High-quality polymorphic SNPs from the parental lines were combined (*in silico*) to build an artificial single-cross genomic matrix. In addition, duplicate markers between chromosomes were removed to avoid overparameterization caused by multicollinearity. Finally, markers with minor allele frequency (MAF)<0.05 were removed from the single-cross genomic matrices, resulting in 11,884 (SNP array), 10,361 (GBS-B73), and 4,801 (GBS-Mock) SNP markers to perform the remaining analysis.

### 2.5 Analysis of population structure and genetic diversity

The three SNP datasets (SNP array, GBS-B73, and GBS-Mock) from the 330 parental lines were used to assess the population structure of the panel. In these analyses, in particular, heterozygous loci and rare variants (MAF< 0.05) were considered to capture all diversity and variability to perform principal component analysis (PCA) and determine the relatedness between parental lines.

K-means clustering was applied, using the total within-cluster sum of square (WSS) method to determine the optimal number of clusters so that the total intra-cluster variation is minimized (Kassambara, 2017). The factoextra package (Kassambara and Mundt, 2020) was used for this. Subsequently, Kendall’s method determined the coincidence in forming clusters among the different datasets (Kendall, 1938). Kendall’s tau correlation coefficient was tested at a probability level of 0.01. PCA was performed, and biplots were constructed to assess population structure.

The genetic distances between the parental lines were calculated for each SNP dataset using the Rogers distance (Rogers, 1972). Subsequently, to measure the correlation among the kinship matrices, the Mantel correlation test (Mantel, 1967) was applied to detect significance. The Mantel correlation test is non-parametric and computes the significance of the correlation similarity measures using 1,000 permutations of the rows and columns of one distance matrix. The heatmaps of the genetic distance matrices were obtained using the *superheat R* package (Barter and Yu, 2018). Correlations were obtained using the *vegan* package (Oksanen et al., 2019), and each analysis was performed for each SNP dataset scenario.

### 2.6 Full diallel genomic analysis

To find out how diversity and population structure can influence the formation of heterotic groups, it was necessary to construct *in silico* genome of the 751 single crosses from parental lines. Therefore, at this stage, we combined phenotypic and genotypic information from these individuals to estimate general (GCA) and specific combining abilities (SCA). For this, the following diallel model was adjusted:


$$y=Z_{P}g_{P}\;+\;Z_{H}h\;+\;\text{ϵ},$$

where ***y*** is the adjusted phenotypic data vector of the single crosses for the trait, g_P_ is the random effects vector of the GCA captured by the markers of the parental lines, and h is the random effects vector of the SCA that denotes the interaction effects across the parental lines. Z_P_ and Z_H_ are incidence matrices that relate *y* to g_P_ and h to g_p_ ~ *N* (0, σ^2^_p_G_p_) and h ~ *N* (0, σ^2^_H_H), where σ^2^_P_ and σ^2^_H_ are variance components associated with GCA and SCA, respectively. G_P_ and H are relationship matrices for the parental lines and single crosses, respectively. Finally, ε ~ *N* (0, σ^2^_ε_I), where σ^2^_ε_ is the variance associated with the residuals.

The G_P_ relationship matrix was calculated using the SNP markers according to (VanRaden, 2008), where W_P_ is the matrix of centered and patterned markers. Therefore, $G_{P}=\frac{W_{p}W_{p}^{'}}{p}$ (Technow et al., 2014; Lopez-Cruz et al., 2015), where *p* is the number of markers. This resulted in an average diagonal G_p_ value of ~1; therefore, σ^2^_p_ was defined on the same scale as σ^2^_ε_. The elements of the H matrix were obtained directly from the G_P_ (Bernardo, 2002; Technow et al., 2014). The matrix H for all possible crosses was obtained with the Kronecker product between G_P_’s, H = G_p_ ⊗ G_p_ (Covarrubias-Pazaran, 2016). A model was built with their respective kernels for each SNP marker source. Analyses were performed using the *ASReml-R* package (Butler et al., 2018).

### 2.7 Heterotic groups and testers

The determination of heterotic groups was performed based on SCA estimates for each trait. These estimates corresponded to a matrix of genetic distances. According to Falconer and Mackay (1996), the genetic distance between parents positively affects heterosis. This association depends on dominance effects or differences in the frequency of the alleles that control the trait considered (Falconer, 1960). Burstin et al. (1994) also found that SCA variance is an indicator for predicting hybrid performance by genetic distance between parents. According to this information, it was assumed that the higher the SCA estimates, the greater the distance between the parents and the more significant the heterosis. From this, the 45 lines were divided into two heterotic groups.

The SCA estimates were submitted to a clustering algorithm, K-means, which grouped them according to the SCA estimates. To estimate the correlation between the heterotic groups formed for the different genotyping methods, Pearson’s correlation was applied and tested at a probability level of 0.01 by Student’s t-test. Subsequently, the identification of the best tester in each group was performed according to the GCA estimates. The best tester of a given group was the line that showed the highest GCA with the other group. Based on this, the coincidence of testers between the scenarios was evaluated.

### 2.8 Obtaining single-cross combinations and genomic prediction

After the parental lines were divided into heterotic groups, only the single crosses corresponding to interpopulation crosses *via* North Carolina II (NCII) design were considered for the following analyses. The number of single crosses changed according to the configuration of heterotic groups for each trait in the three SNP scenarios.

For the genomic prediction of the single crosses, an additive–dominance genomic best linear unbiased prediction (GBLUP) model was used, as described below:


$$\hat{y}=1µ+Za+Zd+\epsilon ,$$

where $\hat{y}$ is the adjusted means vector of the single crosses for the trait; µ is the mean (intercept); a is the vector of additive genetic effects of individuals, where a ~ *N* (0, G_a_σ^2^_a_); d is the vector of dominance effects, where d ~ *N* (0, G_d_σ^2^_d_); ε is the random effects vector of the residuals, where ε ~ *N* (0, I σ^2^_ε_). Z is the incidence matrix for a and d. σ^2^_a_ is the additive genomic variance, σ^2^_d_ is the dominance genomic variance, and σ^2^_ε_ is the residual variance. G_a_ and G_d_ are the additive and dominance genomic relationship matrices, respectively, of the single crosses, where $G_{a}=\frac{W_{A}W_{A}^{'}}{2\sum_{i=1}^{n}p_{i}\left(1-p_{i}\right)}$ and $G_{d}=\frac{W_{D}W_{D}^{'}}{4\sum_{i=1}^{n}{\left(p_{i}\left(1-p_{i}\right)\right)}^{2}}$ , where *p_i_* is the frequency of an allele at locus *i* and W is the matrix incidence of markers (VanRaden, 2008). The W_A_ matrix was encoded as 0 for A_1_A_1_, 1 for A_1_A_2_ heterozygote, and 2 for A_2_A_2_ homozygote. For W_D_, genotypes were coded as 0 for both homozygotes and 1 for the heterozygote. The genomic relationship matrices were built using the *snpReady* package (Granato et al., 2018). The genomic prediction models were performed using the *sommer* package (Covarrubias-Pazaran, 2016). It is worth noting that all three sets of markers were used to build the kernels. The Mantel correlation test (Mantel, 1967) was applied to detect the significance between the additive and dominance genomic relationship matrices.

To evaluate the model performance, we used the CV-α cross-validation with five folds and four replicates (Yassue et al., 2021b). The predictive ability was estimated by Pearson’s correlation between predicted genotypic and observed values from the validation set. The prediction accuracy was estimated by the correlation between predictive ability and heritability, according to Mrode (2014). Correspondence between phenotypic and genotypic selection was calculated for each set of markers through the percentage of common genotypes selected by their adjusted means from the phenotypic analysis and their genomic estimated breeding values (GEBVs) from the genomic prediction model concerning different intensities of selection (1%, 10%, 20%, 30%, and 40%). The heritability in the broad-sense (H^2^) and the narrow-sense (h^2^) was also estimated by the equations below:


$$H^{2}=\frac{{\hat{\sigma }}_{a}^{2}+{\hat{\sigma }}_{d}^{\;2}}{\left({\hat{\sigma }}_{a}^{2}+{\hat{\sigma }}_{d}^{2}+{\hat{\sigma }}_{\text{ϵ}}^{2}\right)}\text{and }h^{2}=\frac{{\hat{\sigma }}_{a}^{2}}{\left({\hat{\sigma }}_{a}^{2}+{\hat{\sigma }}_{d}^{2}+{\hat{\sigma }}_{\text{ϵ}}^{2}\right)},$$

where ${\hat{\sigma }}_{a}^{2}$ is the additive genetic variance, ${\hat{\sigma }}_{d}^{2}$ is the dominance genetic variance, and ${\hat{\sigma }}_{\epsilon }^{2}$ is the residual variance.

## 3 Results

### 3.1 Genetic diversity and population structure

**Figure 2:**
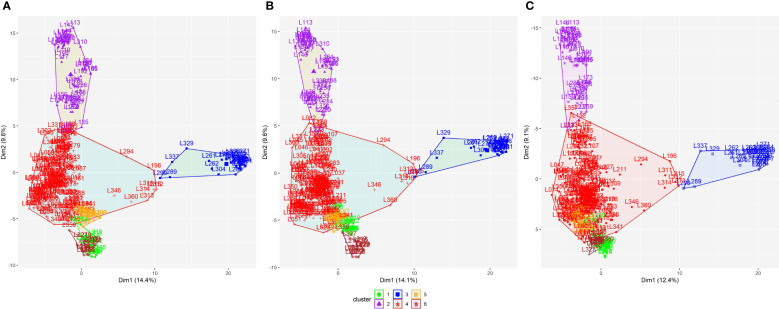
K-means clustering method for dividing the 330 parental lines into subpopulations applied to all SNP datasets: **(A)** SNP array, **(B)** GBS-B73, and **(C)** GBS-Mock. SNP, single-nucleotide polymorphism.

According to the WSS method, for all datasets, the optimal number of clusters among the 330 parental lines that minimized within-group variance and maximized between-group variance was six (**Figure S1**). Subsequently, the K-means clustering method showed a remarkable similarity in the arrangement of clusters among datasets (**Figure 2**). This similarity is confirmed by the coincidence values in the clustering (**Table S1**), with correlation coefficients above 0.95.

Concerning PCA, the SNP datasets showed similar performances regarding the variance explained by the principal components. The first principal components hold the highest percentage of explained variance (**Figure S2A**). When considering the first 10 main components, SNP array showed the highest value of cumulative explained variance (27.3%). At the same time, GBS-B73 and GBS-Mock presented discounts of 24.1% and 16.8%, respectively (**Figure S2B**).

**Figure 3:**
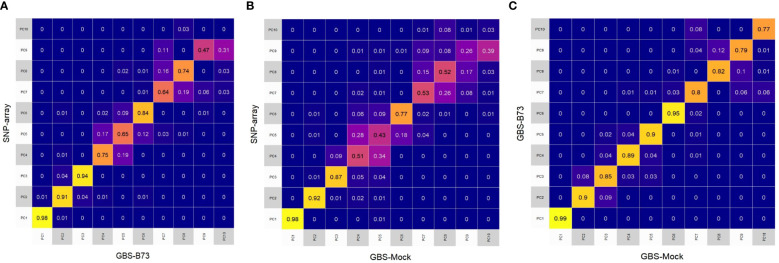
Heatmap of the coefficient of determination (R^2^) of the 10 first eigenvectors built from the Rogers distance among all SNP datasets: **(A)** SNP array and GBS-B73, **(B)** SNP array and GBS-Mock, and **(C)** GBS-B73 and GBS-Mock. SNP, single-nucleotide polymorphism.

In general, PCA revealed that the first eigenvectors exhibited similar patterns of variance in all combinations between datasets, supported by the coefficient of determination (R^2^). However, the other eigenvectors showed less similarity between the captured variance patterns (**Figure 3**). The first four eigenvectors of SNP array and GBS-B73 showed high values of R^2^ (**Figure 3A**). In contrast, for SNP array and GBS-Mock, the highest values of R^2^ were concentrated in the first three eigenvectors (**Figure 3B**). For GBS-B73 and GBS-Mock, all eigenvectors showed high magnitude R^2^, with the former being slightly higher than the others (**Figure 3C**).

**Figure 4:**
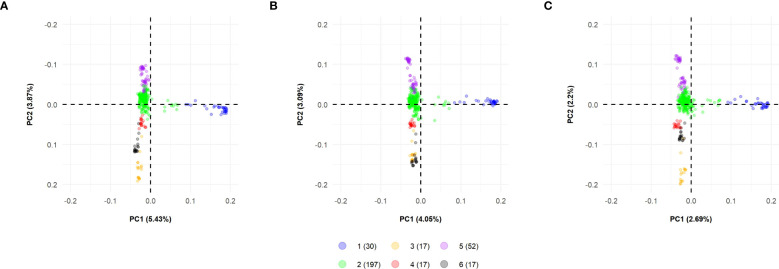
Biplot among two first principal components using all datasets for 330 tropical parental lines: **(A)** SNP array, **(B)** GBS-B73, and **(C)** GBS-Mock. Explained variance percentages of each principal component are in parentheses. Clusters were used to color-code parental lines. SNP, single-nucleotide polymorphism.

Biplots were constructed to visualize the spatial distribution of lines in all SNP datasets (**Figure 4**; **S3**, **S4**). For this, the first three PCs were used, together with the information obtained by the K-means clustering method (**Figure 2**). All datasets showed the same pattern of dispersion among the lines, in agreement with the cluster analysis, which suggests that the SNP datasets capture similar patterns of variance (**Figure 4**).

Rogers distance matrices (GD) from all SNP datasets sampled similar groups and subgroups, with slight differences between them (**Figure S5**). Regarding the Mantel correlations between the GDs, high magnitude correlations (>0.83) were observed involving different scenarios (**Table 1**).

**Table 1:** Mantel correlation of Rogers genetic distance (GD) matrices for 330 parental lines and of additive genomic relationship (*Ga*) and dominance genomic relationship (*Gd*) matrices for 751 maize single crosses estimated from SNP array, GBS-B73, and GBS-Mock markers.

		GBS-B73	GBS-Mock
***GD***	SNP array	0.91**	0.83**
	GBS-B73	–	0.91**
***G_a_***	SNP array	0.97**	0.96**
	GBS-B73	–	0.99**
***G_d_***	SNP array	0.78**	0.58**
	GBS-B73	–	0.72**

Rogers genetic distance (GD) matrices were computed with markers from 330 parental lines data. *Ga* and *Gd* matrices were computed with markers from 751 maize singles-crosses.

SNP array, Affymetrix^®^ Axiom Maize Genotyping array; GBS-B73, genotyping-by-sequence with SNP calling using B73 as reference genome; GBS-Mock, genotyping-by-sequence with SNP calling using the mock reference built with all parental lines.

SNP, single-nucleotide polymorphism.

**Empirical significance level from permutations.

The symbol "-" means that the correlation of a value with itself is maximum, that is "1".

### 3.2 Variance components, genomic heritability, and genomic relationship matrices

Broad- and narrow-sense heritabilities were higher for EH, followed by PH and GY (**Table S2**). GY showed broad-sense heritability for all SNP datasets, on average, 36% higher than narrow-sense heritability. This difference is significantly smaller for the other traits, 15% and 6%, for PH and EH, respectively. The narrow-sense heritability for all SNP datasets was practically the same for GY. As for PH, there was a slight difference in SNP array, and GBS-Mock presented heritability slightly higher than that of GBS-B73. For EH traits, narrow-sense heritability varied little among SNP datasets, with GBS-Mock and SNP array showing the highest heritabilities. The heritabilities in the broad-sense (H^2^) followed the same tendency.

Regarding the additive genomic relationship matrices (*Ga*) across the single-crosses, SNP array, GBS-B73, and GBS-Mock showed high Mantel correlations (**Table 1**; **Figures S6A-C**). However, the genomic dominance relationship matrices (*Gd*) showed lower correlations than *Ga*. The correlations between the dominance relationship matrices (*Gd*) were lower but still from medium to high. GBS-Mock stands out with a correlation of 0.72 with GBS-B73.

### 3.3 Heterotic groups and testers

**Figure 5:**
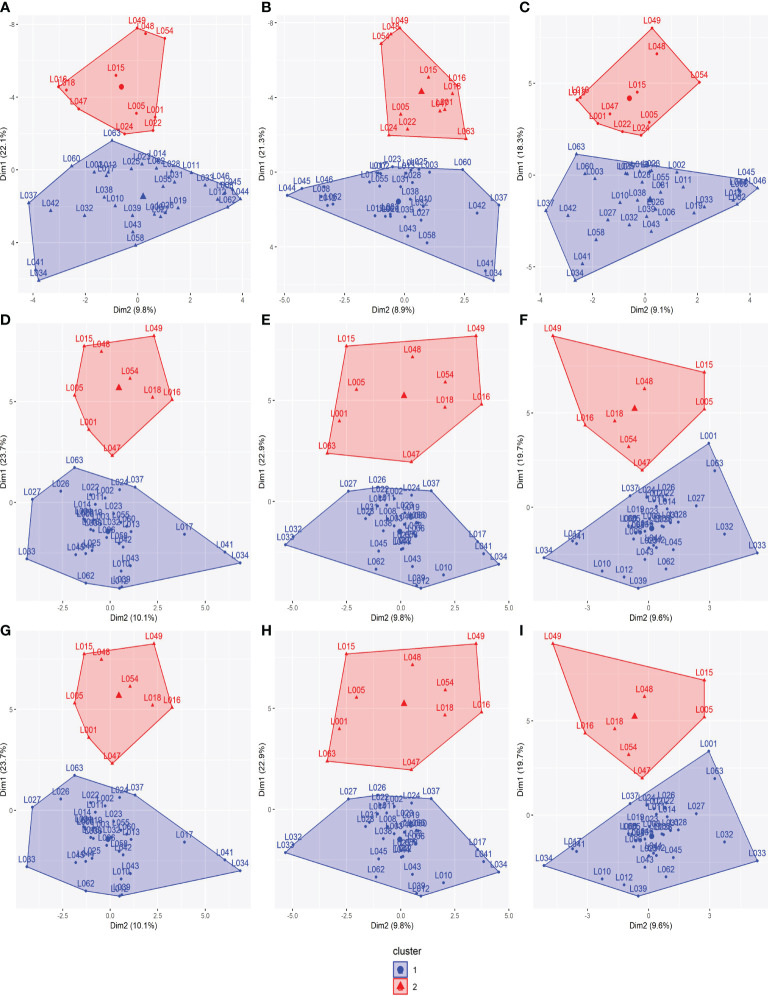
Heterotic groups among the 45 tropical parental lines for all traits: **(A)** SNP array (GY), **(B)** GBS-B73 (GY), **(C)** GBS-Mock (GY), **(D)** SNP array (PH), **(E)** GBS-B73 (PH), **(F)** GBS-Mock (PH), **(G)** SNP array (EH), **(H)** GBS-B73 (EH), and **(I)** GBS-Mock (EH). GY, grain yield; PH, plant height: EH, ear height; SNP, single-nucleotide polymorphism.

Based on SCA estimates, the 45 parental lines were divided into heterotic groups as the genetic distance between them for the evaluated traits, GY, PH, and EH. Accordingly, two heterotic groups were formed for all SNP datasets (**Figure 5**). The formation of heterotic groups among the SNP datasets was quite similar, with high correlations, higher than 0.94 for GY and 0.87 for PH and EH (**Table S3**). There was, at most, a change in the allocation of two parental lines between heterotic groups in different SNP datasets. Likewise, the SCA correlations of the parental lines among the SNP datasets were higher than 0.96 (**Table S4**).

GCA estimates from each parental line, trait, and SNP dataset were used to choose the best tester in each group (**Table 2**). Thus, the testers matched among SNP datasets in the respective heterotic groups for each trait. Based on GY, the tester chosen for heterotic group one (HP_1_) was L023, and for heterotic group two (HP_2_), it was L006. As for PH and EH, L001 was elected as the HP_1_ tester and L003 as the HP_2_ tester. The correlations between the GCAs confirm this result, with maximum correlations (**Table S4**).

**Table 2:** Choice of the best tester according to the SNP datasets (SNP array, GBS-B73, and GBS-Mock), evaluated traits (GY, PH, and EH), and heterotic groups (HP_1_ and HP_2_).

		Testers	
		HP _1_	HP _2_
**GY**	**SNP array**	L023	L006
	**GBS-B73**	L023	L006
	**GBS-Mock**	L023	L006
	**SNP array**	L001	L003
**PH**	**GBS-B73**	L001	L003
	**GBS-Mock**	L001	L003
**EH**	**SNP array**	L001	L003
	**GBS-B73**	L001	L003
	**GBS-Mock**	L001	L003

SNP array: Affymetrix^®^ Axiom Maize Genotyping array: GBS-B73, genotyping-by-sequence with SNP calling using B73 as reference genome; GBS-Mock, genotyping-by-sequence with SNP calling using the mock reference built with all parental lines.

SNP, single-nucleotide polymorphism.

### 3.4 Genomic prediction

**Figure 6:**
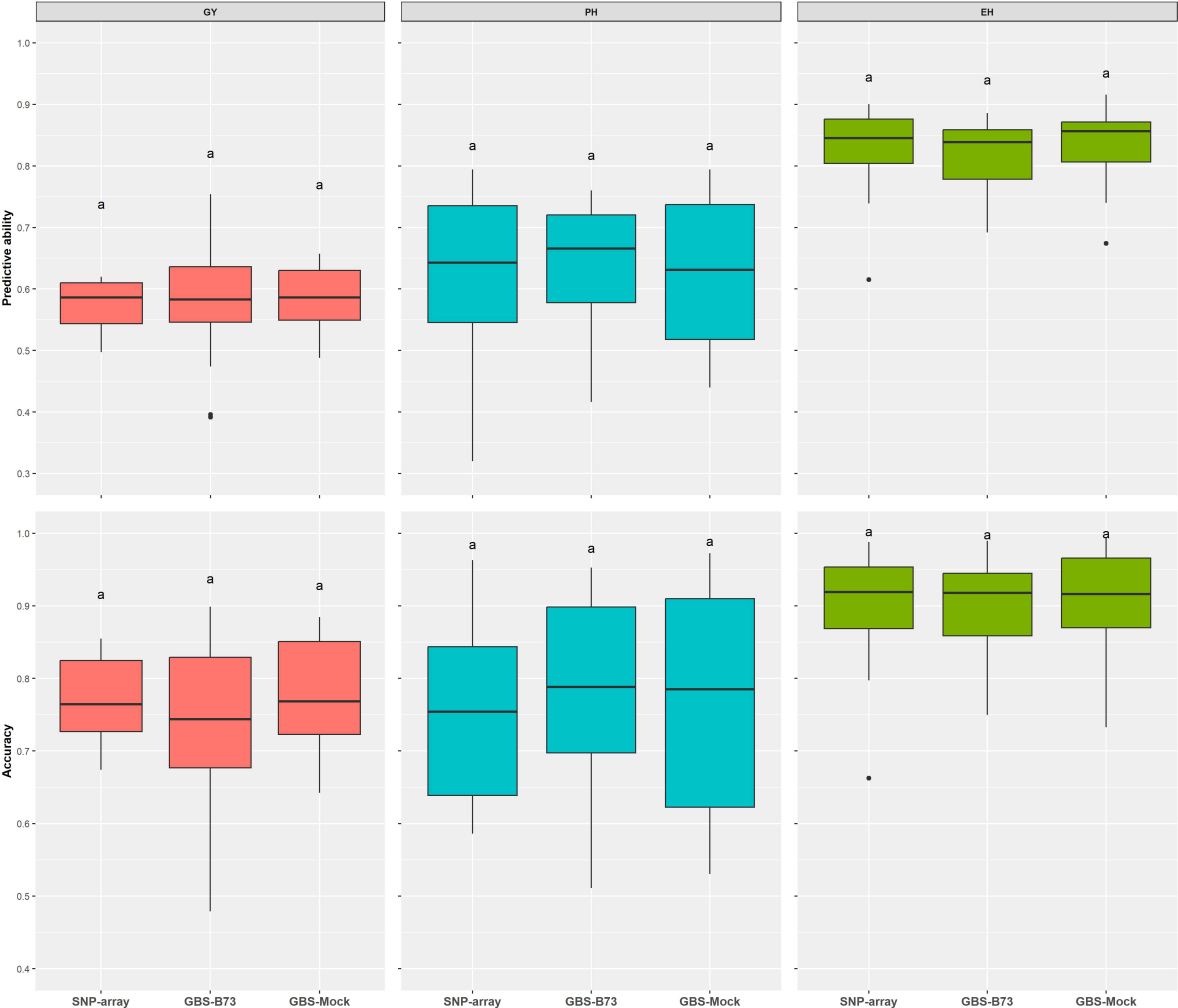
Predictive ability via additive–dominance GBLUP model estimated by Pearson’s correlation between predicted and observed genotypic values of the validation set for all SNP datasets (SNP array, GBS-B73, and GBS-Mock). GBLUP, genomic best linear unbiased prediction; SNP, single-nucleotide polymorphism. Equal letters indicate no significant differences between groups (Tukey's post hoc test, P < 0.05).

**Figure 7:**
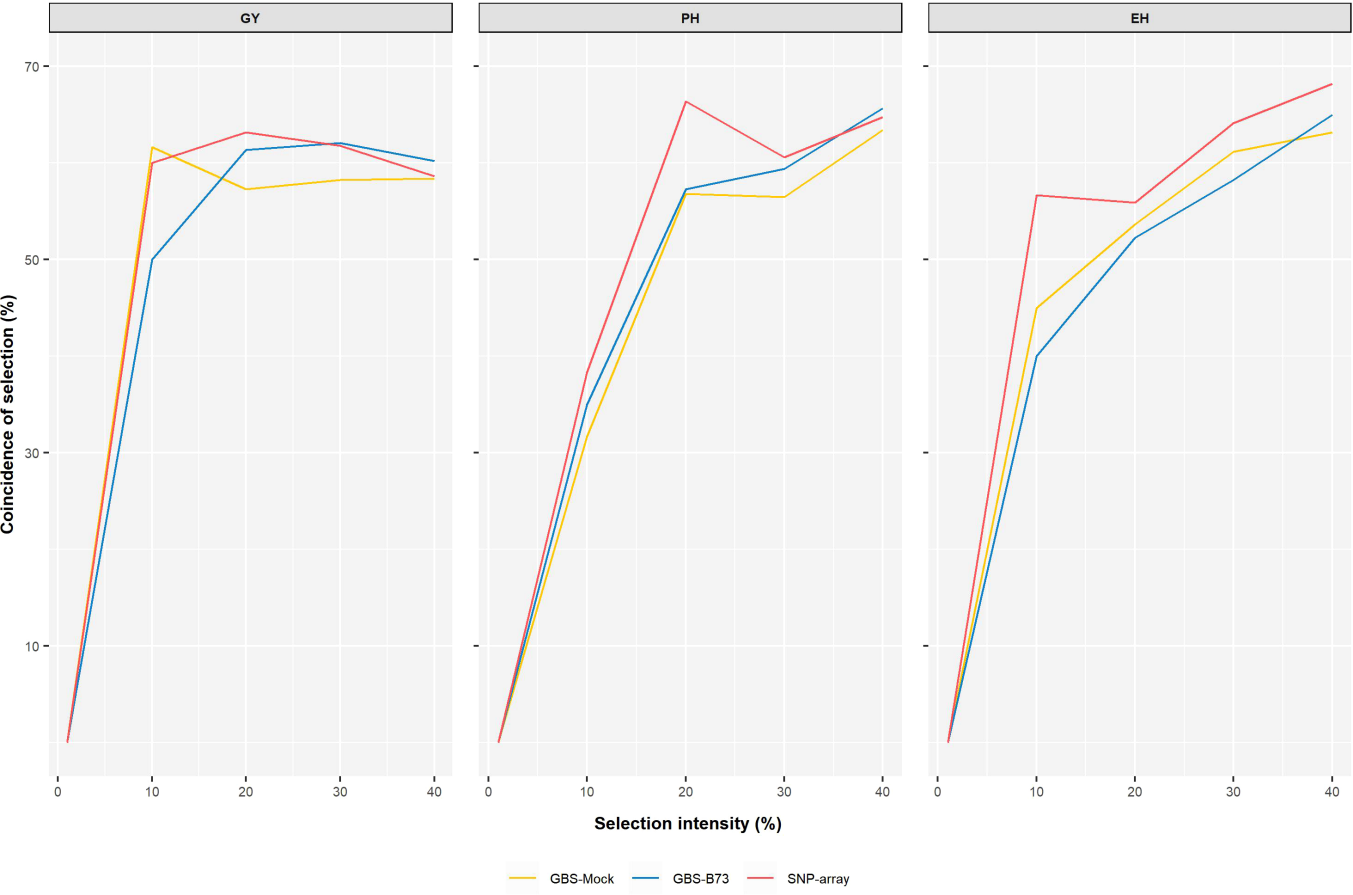
Coincidence between phenotypic and genotypic selection for each set of markers from the genomic prediction model concerning different selection intensities. The coincidence of selection percentage (y-axis) under a series of continuous selection intensities (1%–40%) (x-axis).

The predictive ability estimated by the additive–dominance model for all traits and following the same trend as the other results did not vary significantly among SNP datasets. The mean values of PA were 0.58 for GY, 0.64 for PH, and 0.83 for EH. Prediction accuracy also did not vary significantly between SNP datasets. It showed a high magnitude for all traits, with a mean value of 76% for GY, 78% for PH, and 90% for EH (**Figure 6**). The coincidence between selected individuals based on the adjusted means of the phenotypic analysis and the GEBVs of the genomic prediction model was generally satisfactory. It increased with rising selection intensity (**Figure 7**). Although GY is considered the most complex, the selection coincidence levels of this one were similar to the other traits. SNP array showed slightly higher coincidence values for almost all selection intensities. However, the different datasets showed approximate coincident values.

## 4 Discussion

Recent crop genetics and genomics advances have gained remarkable attention and offered genotyping technologies (Chakradhar et al., 2017). Various genotyping platforms are available to meet the most diverse needs regarding costs per sample and different marker densities (Thomson, 2014). GBS, in particular, has emerged as a cost-effective strategy for genome-wide SNP discovery and population genotyping due to the simple library preparation and the robust approach to genome reduction (Elshire et al., 2011).

All this progress is focused on a small group of crops (Tester and Langridge, 2010) to the detriment of smaller agricultural species, considered orphans, historically poorly researched (Mayes et al., 2012), in that the large majority do not have a reference genome. Sabadin et al. (2022) showed that using mock genomes could be a worthy strategy that permits using SNP markers for genomic selection in orphan crops. However, orphan crop breeding programs focused on single-cross development must also determine heterotic groups to maximize the heterosis. Our study aims to go forward and verify the usefulness of mock genomes as a method to permit reliable heterotic group clustering.

### 4.1 Influence of genotyping methods on population structure and diversity

The study of the characterization of genetic diversity, population structure, and genetic relationships among elite germplasm parents, based on molecular markers, can accelerate genetic gains in breeding programs (Romay et al., 2013; Adu et al., 2019). This study helps understand how the germplasm is organized in selecting parents that present effective contributions and in the designation of heterotic groups (Wu et al., 2016). Thus, genomic data not only allow the estimation of genetic diversity but also combine them with phenotypic information to find new functional genes and build prediction models (Milner et al., 2019). However, in this topic, the focus is on whether, with the simulated reference genome, there is the discovery of the same polymorphisms and how it reflects on the population structure of the lines.

The WSS method indicated the optimal number of clusters by locating a curve on the plot, which is generally considered an indicator of the optimal number of groups (Kassambara, 2017). With this information and the results of the K-means clustering, the parental lines were partitioned into subpopulations, where the SNP datasets showed similar behavior (**Figure S1**; **Figure 2**; **Table S1**), in agreement with the spatial distributions obtained in the biplot graphs (**Figure 4**), in which all SNP datasets showed the same dispersion pattern between lines. This suggests that the SNP datasets capture similar patterns of variance, despite the difference in the number of markers between them, where GBS-Mock has a lower number and the difference in the genotyping platform itself (array and GBS). Thus, SNP array, GBS-B73, and GBS-Mock revealed similar performances concerning genetic diversity and the population structure of parental lines. Darrier et al. (2019) compared the performance of SNP array and GBS to investigate the extent and pattern of genetic variance in barley and observed that the two methodologies selectively access the informative polymorphism in different portions of the genome. Despite this, their results showed a strong positive correlation between the matrices of both genotyping approaches, supporting their similarity and validity.

PCA shows that these variance patterns captured by the SNP datasets are more similar concerning the first eigenvectors (**Figure 3**). However, the captured variance is more consistent when comparing GBS-B73 and GBS-Mock (**Figure 3C**). This can be explained by the verification bias existing in SNP array since this bias arises when the markers are not obtained from a random sample of the polymorphisms of the population of interest since the matrix is ​​constructed using temperate maize lines (Frascaroli et al., 2013; Heslot et al., 2013; Unterseer et al., 2014), and the lines in the study are from tropical germplasm.

The matrices of genetic distances among the parental lines revealed similar patterns, showing the formation of subpopulations between the lines (**Figure S5**). When using wheat as a model species to test for verification bias and investigate its impact on genetic diversity estimates, Chu et al. (2020) observed a tendency for SNP array, leading to an underestimation of molecular diversity within the population. These results agree with a previous study on wheat lines (Elbasyoni et al., 2018) and maize lines (Frascaroli et al., 2013). Despite the verification bias mentioned above and the difference between the reference genome used, the temperate B73 genome, or the mock genome, the population structure between the lines did not show a significant difference, as the correlations between the matrices of genetic distances were of high magnitude. Even though GBS-Mock uses a different reference genome from SNP array and GBS-B73, their correlation was high (**Table 1**). Elbasyoni et al. (2018), investigating the influence of SNPs from different genotyping platforms on genomic prediction, observed a high correlation (r = 0.77) between SNP array and GBS genetic distance matrices. These high-magnitude correlations suggest that the broad sampling of diversity is well represented by the approaches used in the study. This is supported by the GWAS by Darrier et al. (2019). They indicated that SNP array and GBS methods could detect markers closely associated with genes that control key phenotypic traits.

### 4.2 Influence of genotyping methods in the determination of heterotic groups in the choice of testers

Heterosis is a fundamental phenomenon in obtaining superior single crosses. Establishing heterotic groups to exploit them effectively throughout the breeding cycles is necessary. These, in turn, are made up of genetically related parental lines, which generate little or no heterosis when crossed with each other. Crossing with lines from another heterotic group tends to result in vigorous single crosses (Lee, 1995). Therefore, genetic diversity among heterotic groups tends to increase the level of heterosis detected in hybrid combinations (Falconer and Mackay, 1996; Fu et al., 2014). Badu-Apraku et al. (2011) reported in their diallel study between maize lines that their genetic diversity was small, and because of this, distinct heterotic groups could not be identified. Significant genetic diversity was found in a similar study with other maize lines, and two clear heterotic groups were identified. The type of predominant gene action in the parents under investigation is another factor that affects heterotic clustering. When additive and non-additive effects are significant, and there is a predominance of additive gene action over non-additive gene action, heterotic groups are easily identified (Badu-Apraku et al., 2015; Badu-Apraku et al., 2016a; Badu-Apraku et al., 2016b).

The PH and EH traits showed higher proportions of additive variance captured by the *Ga* matrices than GY (**Table S2**). Although these traits have polygenic inheritance, GY is the most complex trait and most influenced by dominance deviations (Fischer et al., 2008; Hallauer et al., 2010). According to Hallauer et al. (2010), most of the loci involved with GY in maize are due to the occurrence of dominance. This is reflected in a greater difference between H^2^ and h^2^ for GY than for the other traits, confirming the greater influence of dominance deviations on this trait. The additive genomic relationship matrices of the single crosses (*Ga*) showed high correlations among SNP array, GBS-B73, and GBS-Mock, indicating that these approaches capture similar additive variance patterns. GBS-Mock captures additive relationships in single crosses similar to standard procedures, SNP array, and GBS-B73 (**Table 1**; **Figures S6A–C**). However, the correlations between the dominance relationship matrices (*Gd*) were lower but still from medium to high. In both *Ga* and *Gd*, the correlations between SNP array and GBS-Mock were lower, which can be explained by the fact that these SNP datasets use different reference genomes to perform SNP calling.

SCA reflects the action of non-additive gene effects, indicating intra-allelic interactions, is one of the most important parameters in identifying superior single crosses, and is an indicator of genetic distance between parents (Sprague and Tatum, 1942; Carvalho, 1993). Thus, using the SCA estimates as the genetic distance between the lines to identify the panel structure, two heterotic groups were formed, in which the distance between them is maximized. The correlations between the SCA estimates were almost perfect (**Table S4**). In other words, SNP array, GBS-B73, and GBS-Mock presented equivalent SCA estimates. Thus, the composition of heterotic groups practically did not change from one SNP dataset to another. Therefore, the determination of heterotic groups was similar regardless of the platform used (**Figure 5**; **Table S3**).

In addition to presenting distinct heterotic groups, a well-established breeding program also offers good testers. When crossed with parental lines, these provide information about the genetic value of the lines when evaluating the ability to combine between them since it is associated with the additive effects of alleles and additive-type epistatic actions (Cruz and Vencovsky, 1989; Albrecht et al., 2014). The correct choice of a tester can have great significance in the expectation of a successful selection process (Miranda Filho, 2018). According to Hallauer and Martinson (1975), a good tester presents simplicity in use, information that correctly classifies the relative merit of the lines, and the potential for maximizing genetic gain. Thus, based on the GCA estimates between the lines, testers were elected for each heterotic group based on the evaluated traits and the SNP datasets. As expected, there were no differences in tester choice between SNP datasets, as the correlations between GCA estimates across rows were perfect (**Table 2**; **Table S5**).

The genotyping approaches produced very similar results but not the same as previous results regarding the study of the population structure of parental lines; it was expected that this would somehow influence the formation of heterotic groups and the choice of testers. However, given the results, the genotyping platform and, more specifically, the approach that uses the simulated genome as a strategy, the GBS-Mock, produced similar results to the standard procedures.

### 4.3 Influence of genotyping methods on genomic prediction of single crosses

Assessing the performance of all single-cross combinations of parental lines that excel in a breeding program is impractical in most cases, given that the number of combinations grows exponentially as the number of elite parents increases. Thus, obtaining estimates of the genetic values of single crosses not evaluated became viable with the increased availability of molecular markers and genomic prediction models (Hallauer et al., 2010). Therefore, to accelerate genetic gain with limited resources, the prediction of single-cross performance is highly important in modern breeding programs (Basnet et al., 2019).

However, few studies still address how genotyping platforms influence single crosses’ prediction and, more specifically, regarding the mock genome as a tool for more sophisticated analyses, such as genomic prediction. Only one recent study shows the mock genome’s efficiency in predicting maize single crosses, which may be an alternative for crops that do not yet have a reference genome (Sabadin et al., 2022). However, our study is more complete and more representative because obtaining approaches from the population structure phase is crucial for the intended use of germplasm through the division of heterotic groups, the definition of testers, and, finally, the genomic prediction of single crosses.

Predictive ability and prediction accuracy are closely related measures. Therefore, we will only discuss it based on predictive ability. GY showed the lowest predictive abilities in all SNP datasets, and EH has the highest in the additive–dominance GBLUP prediction model (**Figure 6**, **Table S2**). Combs and Bernardo (2013) suggested that genomic predictions are more accurate for traits with higher heritability. In the results of Hayes et al. (2010), complex traits controlled by many small effect loci, such as GY, showed lower predictive abilities than less complex traits. Although GBS-Mock has a lower number of markers, this approach presented a similar performance to the other SNP datasets for all traits, corroborating the hypothesis that it is possible to substantially reduce the number of markers and maintain a high predictive ability (Tayeh et al., 2015; Ma et al., 2016; Sousa et al., 2019), except for long-term breeding cycles without updating the training population that would demand high marker densities (DoVale et al., 2022). In addition, the genetic distance estimates between the SNP datasets were very similar (**Figure S5**).

Selection intensity must be chosen thoughtfully, as genetic variability can be drastically reduced with high selection pressure. The choice of appropriate selection intensities depends on the size of the population and the duration of the breeding program, whether short-term or long-term. In general, selection intensities ranging from 10% to 40% are used in plant breeding, the highest being applied at the beginning of a breeding program (Hallauer et al., 2010). For the coincidence of individuals by phenotypic selection and genomic selection, the SNP datasets showed similar behavior as the selection intensity was increased, being more pronounced from 1% to 10% of selection intensity. From then on, observing the coincidence of selection gains smaller increments (**Figure 7**). Our results for predictive ability and coincidence of selection agree with the results of Sabadin et al. (2022). It is valid to consider that those different intensities modify the response rates. Thus, this coincidence between phenotypic and genomic selections is expected to reach a plateau and subsequently decrease.

Despite the apparent differences between SNP datasets, the general message is that these approaches perform comparably in the analyses performed in this study, even accessing different types of genomic sequences. While SNP array is derived from exome capture and therefore focused on coding sequence variation, the GBS data represent a wider diversity survey in genomic regions associated with low levels of DNA methylation, which may also include many genes and gene regulatory regions (Darrier et al., 2019; Negro et al., 2019). However, the physical distribution of markers reveals higher frequencies of SNPs at the gene-rich telomeric ends of each of the chromosomes for both approaches, with this frequency being more pronounced in SNP array (Bayer et al., 2017). The platforms probably capture nearby markers in linkage disequilibrium with quantitative trait loci (QTLs). In this sense, using different platforms can be advantageous, as it allows the identification of additional QTLs.

### 4.4 Possible applications of the mock genome in plant breeding

Until recently, only the main commercial crops benefited from state-of-the-art technologies. However, the development of the GBS platform emerged as an alternative for using such technologies to be viable for orphan crops. Approaches like this can convert orphan crops into crops rich in genomic resources and substantially reduce the breeding process (Varshney et al., 2009; Varshney and May, 2012; Varshney et al., 2012).

Previously, this process was much slower than nowadays. Rice, for example, took almost 20 years to stop being an orphan crop and become a basic model for cereals (Varshney et al., 2009). Introducing these crops into the genomic era also accelerates the identification of genes underlying important agronomic traits and improves our understanding of the evolution of these species (Ye and Fan, 2021). However, many minor crops are becoming rich in genetic resources as a result of investments from various public and private initiatives, such as the African Orphan Crops Consortium (AOCC) (Hendre et al., 2019), which is a global partnership that is generating resources genomics for 101 African orphans. One of the objectives of this Consortium is to create reference genomes for these cultures. Although some efforts are being made to pay greater attention to these crops (Chiurugwi et al., 2019; Gregory et al., 2019; Jamnadass et al., 2020), the ideal is still far from being achieved with a view to several species relevant to local diets around the world that are understudied.

Despite initiatives and investments, not all crops will benefit, so they cannot take advantage of modern breeding tools. While these advances are being consolidated, mock genomes can be an alternative, where the absence of a reference genome presents a barrier to the efficient use of GBS data (Melo et al., 2017; Hale et al., 2018). In the meantime, the present study has shown that using a population-tailored mock reference to perform SNP discovery is a valid alternative. With this approach, it was possible to carry out investigations to outline a breeding program, from studies of diversity and population to genomic prediction studies. However, it is important to emphasize that a population with maximum representativeness must be considered when building a mock reference to capture all the population polymorphisms (Sabadin et al., 2022).

These advantages of using a mock genome in genomic studies must consider some caveats; for example, diploid crops with smaller genomes are preferred over cross-pollinated or polyploid orphan crops, as they have genomes that are too complex to be sequenced. However, genome size will become less of a barrier with advances in sequencing technologies and bioinformatics tools (Armstead et al., 2009). Another challenge is in the SNP calling due to the limitations of GBS, which can lead to incorrect identification of homozygotes and heterozygotes because of the low coverage of NGS reads, in addition to a large number of lost and low-quality data (Heslot et al., 2013). According to Sabadin et al. (2022), the mock genomes do not present the physical position of the markers in a constant reference, which hinders studies such as GWAS and candidate gene discovery. Negro et al. (2019) stated that SNP array and GBS are complementary to detect QTLs tagging different haplotypes in association studies. In this sense, using other platforms can be advantageous, as it allows the identification of additional QTLs. However, no studies still demonstrate the performance of mock genomes for these purposes. When looking for these larger effect marks, the results will probably differ from those obtained with SNP array due to changes in coverage between platforms.

Given what has been shown, it is possible to infer and recommend that a mock genome constructed from the population’s polymorphisms to perform the SNP calling is an excellent strategy to support plant breeders in studies of diversity, population structure, the definition of heterotic groups, choice of testers, and genomic prediction in species that still do not have a reference genome available, which is an alternative for the rapid advancement of orphan crop improvement. This approach will play a key role in improving the genetic potential of orphan crops and helping develop sustainable food systems.

## Data availability statement

The datasets presented in this study can be found in online repositories. The names of the repository/repositories and accession number(s) can be found below: https://data.mendeley.com/datasets/5rtc89t7v5/1, DOI:10.17632/5rtc89t7v5.1.

## Author contributions

IM was responsible for writing the report, analysing data, interpreting results, and creating tables/figures. JD was responsible for supervising and analyzing data regarding genomic prediction. FS contributed to the manipulation of the raw genomic data and mock genome assembly. RF-N contributed extracting data and designed the work that led to the submission. JD, FS and RF-N played an important role in interpreting the results and contributed to the report review. All authors contributed to the article and approved the submitted version.

## References

[B1] Adhikari L. Lindstrom O. M. Markham J. Missaoui A. M. (2018). Dissecting key adaptation traits in the polyploid perennial medicago sativa using GBS-SNP mapping. Front. Plant Sci. 9. doi: 10.3389/fpls.2018.00934 PMC6039623 30022989

[B2] Adu G. B. Badu-Apraku B. Akromah R. Garcia-Oliveira A. L. Awuku F. J. Gedil M. (2019). Genetic diversity and population structure of early-maturing tropical maize inbred lines using SNP markers. PloS One 14. doi: 10.1371/journal.pone.0214810 PMC6456193 30964890

[B3] Albrecht J. Gertrud Berens D. Jaroszewicz B. Selva N. Brandl R. Farwig N. (2014). Correlated loss of ecosystem services in coupled mutualistic networks. Nat. Commun. 5. doi: 10.1038/ncomms4810 24806612

[B4] Armstead I. Huang L. Ravagnani A. Robson P. Ougham H. (2009). Bioinformatics in the orphan crops. Brief Bioinform. 10, 645–653. doi: 10.1093/bib/bbp036 19734255

[B5] Bachlava E. Taylor C. A. Tang S. Bowers J. E. Mandel J. R. Burke J. M.. (2012). Snp discovery and development of a high-density genotyping array for sunflower. PloS One 7. doi: 10.1371/journal.pone.0029814 PMC3251610 22238659

[B6] Badu-Apraku B. Fakorede M. A. B. Gedil M. Annor B. Talabi A. O. Akaogu I. C.. (2016a). Heterotic patterns of IITA and CIMMYT early-maturing yellow maize inbreds under contrasting environments. Agron. J. 108, 1321–1336. doi: 10.2134/agronj2015.0425

[B7] Badu-Apraku B. Fakorede M. A. B. Gedil M. Talabi A. O. Annor B. Oyekunle M.. (2015). Heterotic responses among crosses of IITA and CIMMYT early white maize inbred lines under multiple stress environments. Euphytica 206, 245–262. doi: 10.1007/s10681-015-1506-0

[B8] Badu-Apraku B. Fakorede M. A. B. Talabi A. O. Oyekunle M. Akaogu I. C. Akinwale R. O.. (2016b). Gene action and heterotic groups of early white quality protein maize inbreds under multiple stress environments. Crop Sci. 56, 183–199. doi: 10.2135/cropsci2015.05.0276

[B9] Badu-Apraku B. Fontem L. A. Akinwale R. O. Oyekunle M. (2011). Biplot analysis of diallel crosses of early maturing tropical yellow maize inbreds in stress and nonstress environments. Crop Sci. 51, 173–188. doi: 10.2135/cropsci2010.06.0366

[B10] Baldermann S. Blagojević L. Frede K. Klopsch R. Neugart S. Neumann A.. (2016). Are neglected plants the food for the future? CRC Crit. Rev. Plant Sci. 35, 106–119. doi: 10.1080/07352689.2016.1201399

[B11] Barter R. L. Yu B. (2018). Superheat: an r package for creating beautiful and extendable heatmaps for visualizing complex data. J. Comput. Graphical Stat 27, 910–922. doi: 10.1080/10618600.2018.1473780 PMC6430237 30911216

[B12] Basnet B. R. Crossa J. Dreisigacker S. Pérez-Rodríguez P. Manes Y. Singh R. P.. (2019). Hybrid wheat prediction using genomic, pedigree, and environmental covariables interaction models. Plant Genome 12, 180051. doi: 10.3835/plantgenome2018.07.0051 PMC12810112 30951082

[B13] Bayer M. M. Rapazote-Flores P. Ganal M. Hedley P. E. Macaulay M. Plieske J.. (2017). Development and evaluation of a barley 50k iSelect SNP array. Front. Plant Sci. 8. doi: 10.3389/fpls.2017.01792 PMC5651081 29089957

[B14] Bernardo R. (2002). Breeding for quantitative traits in plants (Woodbury: Stemma Press).

[B15] Bevan M. W. Uauy C. (2013). Genomics reveals new landscapes for crop improvement. Genome Biol 14, 206. doi: 10.1186/gb-2013-14-6-206 23796126 PMC3706852

[B16] Browning B. L. Zhou Y. Browning S. R. (2018). A one-penny imputed genome from next-generation reference panels. Am. J. Hum. Genet. 103, 338–348. doi: 10.1016/j.ajhg.2018.07.015 30100085 PMC6128308

[B17] Burstin J. Vienne D. Dubreuil P. Damerval C. (1994). Molecular markers and protein quantities as genetic descriptors in maize. I. Genetic diversity among 21 inbred lines. theoret. app. Genetics 89, 943–950. doi: 10.1007/BF00224522 24178108

[B18] Butler D. G. Cullis B. R. Gilmour A. R. Gogel B. J. Thompson R. (2018) ASReml-r reference manual version 4 ASReml estimates variance components under a general linear mixed model by residual maximum likelihood (REML). Available at: http://www.vsni.co.uk/.

[B19] Carvalho G. R. (1993). Evolutionary aspects of fish distribution: genetic variability and adaptation. J. Fish Biol. 43, 53–73. doi: 10.1111/j.1095-8649.1993.tb01179.x

[B20] Chakradhar T. Hindu V. Reddy P. S. (2017). Genomic-based-breeding tools for tropical maize improvement. Genetica 145, 525–539. doi: 10.1007/s10709-017-9981-y 28875394

[B21] Chiurugwi T. Kemp S. Powell W. Hickey L. T. (2019). Speed breeding orphan crops. Theor. Appl. Genet. 132, 607–616. doi: 10.1007/s00122-018-3202-7 30341490

[B22] Chu J. Zhao Y. Beier S. Schulthess A. W. Stein N. Philipp N.. (2020). Suitability of single-nucleotide polymorphism arrays versus genotyping-By-Sequencing for genebank genomics in wheat. Front. Plant Sci. 11. doi: 10.3389/fpls.2020.00042 PMC7033508 32117381

[B23] Combs E. Bernardo R. (2013). Accuracy of genomewide selection for different traits with constant population size, heritability, and number of markers. Plant Genome 6. doi: 10.3835/plantgenome2012.11.0030

[B24] Covarrubias-Pazaran G. (2016). Genome-assisted prediction of quantitative traits using the r package sommer. PloS One 11. doi: 10.1371/journal.pone.0156744 PMC4894563 27271781

[B25] Crossa J. Beyene Y. Semagn K. Pérez P. Hickey J. M. Chen C.. (2013). Genomic prediction in maize breeding populations with genotyping-by-sequencing. G3: Genes Genomes Genet. 3, 1903–1926. doi: 10.1534/g3.113.008227 PMC3815055 24022750

[B26] Cruz C. D. Vencovsky R. (1989). Comparação de alguns metodos de análise dialélica. Rev. Bras. Genética 12, 425–438.

[B27] Darrier B. Russell J. Milner S. G. Hedley P. E. Shaw P. D. Macaulay M.. (2019). A comparison of mainstream genotyping platforms for the evaluation and use of barley genetic resources. Front. Plant Sci. 10. doi: 10.3389/fpls.2019.00544 PMC6499090 31105733

[B28] DoVale J. C. Carvalho H. F. Sabadin F. Fritsche-Neto R. (2022). Genotyping marker density and prediction models effects in long-term breeding schemes of cross-pollinated crops. Theor. Appl. Genet. 135, 4523–4539. doi: 10.1007/s00122-022-04236-3 36261658

[B29] Elbasyoni I. S. Lorenz A. J. Guttieri M. Frels K. Baenziger P. S. Poland J.. (2018). A comparison between genotyping-by-sequencing and array-based scoring of SNPs for genomic prediction accuracy in winter wheat. Plant Sci. 270, 123–130. doi: 10.1016/j.plantsci.2018.02.019 29576064

[B30] Elshire R. J. Glaubitz J. C. Sun Q. Poland J. A. Kawamoto K. Buckler E. S.. (2011). A robust, simple genotyping-by-sequencing (GBS) approach for high diversity species. PloS One 6. doi: 10.1371/journal.pone.0019379 PMC3087801 21573248

[B31] Falconer D. S. (1960). Introduction to quantitative genetics (Edinburgh: Oliver and Boyd Ltd).

[B32] Falconer D. S. Mackay T. F. C. (1996). Introduction to quantitative genetics. 4th ed (Harlow: Addison Wesley Longman).

[B33] Fischer S. Möhring J. Schön C. C. Piepho H. P. Klein D. Schipprack W.. (2008). Trends in genetic variance components during 30 years of hybrid maize breeding at the university of hohenheim. Plant Breed. 127, 446–451. doi: 10.1111/j.1439-0523.2007.01475.x

[B34] Frascaroli E. Schrag T. A. Melchinger A. E. (2013). Genetic diversity analysis of elite European maize (Zea mays l.) inbred lines using AFLP, SSR, and SNP markers reveals ascertainment bias for a subset of SNPs. Theor. Appl. Genet. 126, 133–141. doi: 10.1007/s00122-012-1968-6 22945268

[B35] Fritsche-Neto R. Akdemir D. Jannink J. L. (2018). Accuracy of genomic selection to predict maize single-crosses obtained through different mating designs. Theor. Appl. Genet. 131, 1153–1162. doi: 10.1007/s00122-018-3068-8 29445844

[B36] Fritsche-Neto R. Galli G. Borges K. L. R. Costa-Neto G. Alves F. C. Sabadin F.. (2021). Optimizing genomic-enabled prediction in small-scale maize hybrid breeding programs: a roadmap review. Front. Plant Sci. 12. doi: 10.3389/fpls.2021.658267 PMC8281958 34276721

[B37] Fritsche-Neto R. Galli G. Mendonça L. de F. Vidotti M. S. Matias F. I.. (2019). USP Tropical maize hybrid panel. Mendeley Data 3.

[B38] Fu D. Xiao M. Hayward A. Fu Y. Liu G. Jiang G.. (2014). Utilization of crop heterosis: a review. Euphytica 197, 161–173. doi: 10.1007/s10681-014-1103-7

[B39] Galli G. Alves F. C. Morosini J. S. Fritsche-Neto R. (2020). On the usefulness of parental lines GWAS for predicting low heritability traits in tropical maize hybrids. PloS One 15. doi: 10.1371/journal.pone.0228724 PMC7006934 32032385

[B40] Ganal M. W. Durstewitz G. Polley A. Bérard A. Buckler E. S. Charcosset A.. (2011). A large maize (zea mays l.) SNP genotyping array: development and germplasm genotyping, and genetic mapping to compare with the B73 reference genome. PloS One 6. doi: 10.1371/journal.pone.0028334 PMC3234264 22174790

[B41] Glaubitz J. C. Casstevens T. M. Lu F. Harriman J. Elshire R. J. Sun Q.. (2014). TASSEL-GBS: a high capacity genotyping by sequencing analysis pipeline. PloS One 9. doi: 10.1371/journal.pone.0090346 PMC3938676 24587335

[B42] Granato I. S. C. Galli G. de Oliveira Couto E. G. e Souza M. B. Mendonça L. F. Fritsche-Neto R. (2018). snpReady: a tool to assist breeders in genomic analysis. Mol. Breed. 38. doi: 10.1007/s11032-018-0844-8

[B43] Gregory P. J. Mayes S. Hui C. H. Jahanshiri E. Julkifle A. Kuppusamy G.. (2019). Crops for the future (CFF): an overview of research efforts in the adoption of underutilised species. Planta 250, 979–988. doi: 10.1007/s00425-019-03179-2 31250097

[B44] Gupta P. K. Rustgi S. Mir R. R. (2008). Array-based high-throughput DNA markers for crop improvement. Heredity (Edinb) 101, 5–18. doi: 10.1038/hdy.2008.35 18461083

[B45] Hale I. Melo A. T. O. Gustafson H. (2018). Sex-linked molecular markers for two cold-hardy kiwifruit species, actinidia arguta and a. kolomikta. Eur. J. Hortic. Sci. 83, 236–246. doi: 10.17660/eJHS.2018/83.4.4

[B46] Hallauer A. R. Carena M. J. Filho J. B. M. (2010). Quantitative Genetics in Maize Breeding. In Handbook of Plant Breeding, vol 6. New York, NY: Springer. doi: 10.1007/978-1-4419-0766-0_1

[B47] Hallauer A. R. Martinson C. A. (1975). Maternal effects in maize hybrids infected with bipolaris maydis (Nisikado) shoemaker, race T 1. Crop Sci. 15, 686–689. doi: 10.2135/cropsci1975.0011183x001500050021x

[B48] Hayes B. J. Pryce J. Chamberlain A. J. Bowman P. J. Goddard M. E. (2010). Genetic architecture of complex traits and accuracy of genomic prediction: coat colour, milk-fat percentage, and type in holstein cattle as contrasting model traits. PloS Genet. 6. doi: 10.1371/journal.pgen.1001139 PMC2944788 20927186

[B49] Hendre P. S. Muthemba S. Kariba R. Muchugi A. Fu Y. Chang Y.. (2019). African Orphan crops consortium (AOCC): status of developing genomic resources for African orphan crops. Planta 250, 989–1003. doi: 10.1007/s00425-019-03156-9 31073657

[B50] Heslot N. Rutkoski J. Poland J. Jannink J. L. Sorrells M. E. (2013). Impact of marker ascertainment bias on genomic selection accuracy and estimates of genetic diversity. PloS One 8. doi: 10.1371/journal.pone.0074612 PMC3764096 24040295

[B51] Holloway H. M. C. P. Yu X. Dunne J. C. Schwartz B. M. Patton A. J. Arellano C.. (2018). A SNP-based high-density linkage map of zoysiagrass (Zoysia japonica steud.) and its use for the identification of QTL associated with winter hardiness. Mol. Breed. 38. doi: 10.1007/s11032-017-0763-0

[B52] Jamnadass R. Mumm R. H. Hale I. Hendre P. Muchugi A. Dawson I. K.. (2020). Enhancing African orphan crops with genomics. Nat. Genet. 52, 356–360. doi: 10.1038/s41588-020-0601-x 32203464

[B53] Kassambara A. (2017) Multivariate analysis I practical guide to cluster analysis in r unsupervised machine learning. Available at: http://www.sthda.com.

[B54] Kassambara A. Mundt F. (2020). Extract and Visualize the Results of Multivariate Data Analyses. Package “factoextra” [WWW Document]. URL http://www.sthda.com/english/rpkgs/factoextra. (Accessed 11.12.23).

[B55] Kendall M. G. (1938). A new measure of rank correlation. Biometrika 30, 81–93. doi: 10.1093/biomet/30.1-2.81

[B56] Lee M. (1995). Dna markers and plant breeding programs. Adv. Agronomy 55, 265–344. doi: 10.1016/S0065-2113(08)60542-8

[B57] Lee Y. G. Jeong N. Kim J. H. Lee K. Kim K. H. Pirani A.. (2015). Development, validation and genetic analysis of a large soybean SNP genotyping array. Plant J. 81, 625–636. doi: 10.1111/tpj.12755 25641104

[B58] Li H. Durbin R. (2009). Fast and accurate short read alignment with burrows-wheeler transform. Bioinformatics 25, 1754–1760. doi: 10.1093/bioinformatics/btp324 19451168 PMC2705234

[B59] Li C. Li Y. Bradbury P. J. Wu X. Shi Y. Song Y.. (2015). Construction of high-quality recombination maps with low-coverage genomic sequencing for joint linkage analysis in maize. BMC Biol. 13. doi: 10.1186/s12915-015-0187-4 PMC4578237 26390990

[B60] Lopez-Cruz M. Crossa J. Bonnett D. Dreisigacker S. Poland J. Jannink J. L.. (2015). Increased prediction accuracy in wheat breeding trials using a marker × environment interaction genomic selection model. G3: Genes Genomes Genet. 5, 569–582. doi: 10.1534/g3.114.016097 PMC4390573 25660166

[B61] Ma Y. Reif J. C. Jiang Y. Wen Z. Wang D. Liu Z.. (2016). Potential of marker selection to increase prediction accuracy of genomic selection in soybean (Glycine max l.). Mol. Breed. 36. doi: 10.1007/s11032-016-0504-9 PMC4965486 27524935

[B62] Mammadov J. Aggarwal R. Buyyarapu R. Kumpatla S. (2012). SNP markers and their impact on plant breeding. Int. J. Plant Genomics 2012. doi: 10.1155/2012/728398 PMC3536327 23316221

[B63] Mantel N. (1967). The detection of disease clustering and a generalized regression approach. Cancer Res. 27, 209–220. 6018555

[B64] Matias F. I. Xavier Meireles K. G. Nagamatsu S. T. Lima Barrios S. C. Borges do Valle C. Carazzolle M. F.. (2019). Expected genotype quality and diploidized marker data from genotyping-by-Sequencing of urochloa spp. tetraploids. Plant Genome 12, 190002. doi: 10.3835/plantgenome2019.01.0002 PMC12810024 33016594

[B65] Mayes S. Massawe F. J. Alderson P. G. Roberts J. A. Azam-Ali S. N. Hermann M. (2012). The potential for underutilized crops to improve security of food production. J. Exp. Bot. 63, 1075–1079. doi: 10.1093/jxb/err396 22131158

[B66] Melo A. T. O. Bartaula R. Hale I. (2016). GBS-SNP-CROP: a reference-optional pipeline for SNP discovery and plant germplasm characterization using variable length, paired-end genotyping-by-sequencing data. BMC Bioinf. 17. doi: 10.1186/s12859-016-0879-y PMC4709900 26754002

[B67] Melo A. T. O. Guthrie R. S. Hale I. (2017). GBS-based deconvolution of the surviving north American collection of cold-hardy kiwifruit (Actinidia spp.) germplasm. PloS One 12. doi: 10.1371/journal.pone.0170580 PMC5268759 28125645

[B68] Mendonça L. de F. Granato Í.S.C. Alves F. C. Morais P. P. P. Vidotti M. S.. (2017). Accuracy and simultaneous selection gains for n-stress tolerance and n-use efficiency in maize tropical lines. Sci. Agric. 74, 481–488. doi: 10.1590/1678-992x-2016-0313

[B69] Messing J. Dooner H. K. (2006). Organization and variability of the maize genome. Curr. Opin. Plant Biol. 9, 157–163. doi: 10.1016/j.pbi.2006.01.009 16459130

[B70] Milner S. G. Jost M. Taketa S. Mazón E. R. Himmelbach A. Oppermann M.. (2019). Genebank genomics highlights the diversity of a global barley collection. Nat. Genet. 51, 319–326. doi: 10.1038/s41588-018-0266-x 30420647

[B71] Miranda Filho J. B. (2018). “Testadores e dialelo,” in Melhoramento de milho. Eds. DeLIMA R. BORÉM A. (Viçosa, MG: Editora UFV), 130–158.

[B72] Mrode R. A. (2014). Linear models for the prediction of animal breeding values. 3rd ed (Edinburgh, UK: CABI).

[B73] Mulvaney M. J. Devkota P. J. (2020). Adjusting crop yield to a standard moisture content. EDIS 2020. doi: 10.32473/edis-ag442-2020

[B74] Munjal G. Hao J. Teuber L. R. Brummer E. C. (2018). Selection mapping identifies loci underpinning autumn dormancy in alfalfa (Medicago sativa). G3: Genes Genomes Genet. 8, 461–468. doi: 10.1534/g3.117.300099 PMC5919736 29255116

[B75] Negro S. S. Millet E. J. Madur D. Bauland C. Combes V. Welcker C.. (2019). Genotyping-by-sequencing and SNP-arrays are complementary for detecting quantitative trait loci by tagging different haplotypes in association studies. BMC Plant Biol. 19. doi: 10.1186/s12870-019-1926-4 PMC6636005 31311506

[B76] Oksanen J. Simpson G. L. Blanchet F. G. Kindt R. Legendre P. Minchin P. R.. (2019) Vegan: community ecology package. Available at: https://github.com/vegandevs/vegan (Accessed November 2, 2022).

[B77] Poland J. A. Brown P. J. Sorrells M. E. Jannink J. L. (2012). Development of high-density genetic maps for barley and wheat using a novel two-enzyme genotyping-by-sequencing approach. PloS One 7. doi: 10.1371/journal.pone.0032253 PMC3289635 22389690

[B78] Rasheed A. Hao Y. Xia X. Khan A. Xu Y. Varshney R. K.. (2017). Crop breeding chips and genotyping platforms: progress, challenges, and perspectives. Mol. Plant 10, 1047–1064. doi: 10.1016/j.molp.2017.06.008 28669791

[B79] Rogers J. S. (1972). “Measures of genetic similarity and genetic distance,” in Studies in genetics VII, vol. 7213. (Texas, USA: University of Texas Publication), 145–153.

[B80] Romay M. C. Millard M. J. Glaubitz J. C. Peiffer J. A. Swarts K. L. Casstevens T. M.. (2013). Comprehensive genotyping of the USA national maize inbred seed bank. Genome Biol. 14. doi: 10.1186/gb-2013-14-6-r55 PMC3707059 23759205

[B81] Sabadin F. Carvalho H. F. Galli G. Fritsche-Neto R. (2022). Population-tailored mock genome enables genomic studies in species without a reference genome. Mol. Genet. Genomics 297, 33–46. doi: 10.1007/s00438-021-01831-9 34755217

[B82] Schnable P. S. Ware D. Fulton R. S. Stein J. C. Wei F. Pasternak S.. (2009). The B73 maize genome: complexity, diversity, and dynamics. Sci. (1979) 326, 1112–1115. doi: 10.1126/science.1178534 19965430

[B83] Singh N. Jayaswal P. K. Panda K. Mandal P. Kumar V. Singh B.. (2015). Single-copy gene based 50 K SNP chip for genetic studies and molecular breeding in rice. Sci. Rep. 5. doi: 10.1038/srep11600 PMC4481378 26111882

[B84] Sousa M. B. Galli G. Lyra D. H. Granato Í.S.C. Matias F. I. Alves F. C.. (2019). Increasing accuracy and reducing costs of genomic prediction by marker selection. Euphytica 215. doi: 10.1007/s10681-019-2339-z

[B85] Sprague G. F. Tatum L. A. (1942). General vs combining ability in single crosses of corn. Agron. J. 34, 923–932. doi: 10.2134/agronj1942.00021962003400100008x

[B86] Tadele Z. Assefa K. (2012). Increasing food production in africa by boosting the productivity of understudied crops. Agronomy 2, 240–283. doi: 10.3390/agronomy2040240

[B87] Tayeh N. Klein A. le Paslier M. C. Jacquin F. Houtin H. Rond C.. (2015). Genomic prediction in pea: effect of marker density and training population size and composition on prediction accuracy. Front. Plant Sci. 6. doi: 10.3389/fpls.2015.00941 PMC4648083 26635819

[B88] Technow F. Schrag T. A. Schipprack W. Bauer E. Simianer H. Melchinger A. E. (2014). Genome properties and prospects of genomic prediction of hybrid performance in a breeding program of maize. Genetics 197, 1343–1355. doi: 10.1534/genetics.114.165860 24850820 PMC4125404

[B89] Tester M. Langridge P. (2010). Breeding technologies to increase crop production in a changing world. Sci. (1979) 327, 818–822. doi: 10.1126/science.1183700 20150489

[B90] Thomson M. J. (2014). High-throughput SNP genotyping to accelerate crop improvement. Plant Breed Biotechnol. 2, 195–212. doi: 10.9787/pbb.2014.2.3.195

[B91] Unterseer S. Bauer E. Haberer G. Seidel M. Knaak C. Ouzunova M.. (2014). A powerful tool for genome analysis in maize: development and evaluation of the high density 600 k SNP genotyping array. BMC Genomics 15. doi: 10.1186/1471-2164-15-823 PMC4192734 25266061

[B92] VanRaden P. M. (2008). Efficient methods to compute genomic predictions. J. Dairy Sci. 91, 4414–4423. doi: 10.3168/jds.2007-0980 18946147

[B93] Varshney R. K. Close T. J. Singh N. K. Hoisington D. A. Cook D. R. (2009). Orphan legume crops enter the genomics era! Curr. Opin. Plant Biol. 12, 202–210. doi: 10.1016/j.pbi.2008.12.004 19157958

[B94] Varshney R. K. May G. D. (2012). Next-generation sequencing technologies: opportunities and obligations in plant genomics. Brief Funct. Genomics 11, 1–2. doi: 10.1093/bfgp/els001 22345600

[B95] Varshney R. K. Ribaut J.-M. Buckler E. S. Tuberosa R. Rafalski J. A. Langridge P. (2012). Can genomics boost productivity of orphan crops? Nat. Biotechnol. 30, 1172–1176. doi: 10.1038/nbt.2440 23222781

[B96] Wang N. Yuan Y. Wang H. Yu D. Liu Y. Zhang A.. (2020). Applications of genotyping-by-sequencing (GBS) in maize genetics and breeding. Sci. Rep. 10. doi: 10.1038/s41598-020-73321-8 PMC7530987 33004874

[B97] Winfield M. O. Allen A. M. Burridge A. J. Barker G. L. A. Benbow H. R. Wilkinson P. A.. (2016). High-density SNP genotyping array for hexaploid wheat and its secondary and tertiary gene pool. Plant Biotechnol. J. 14, 1195–1206. doi: 10.1111/pbi.12485 26466852 PMC4950041

[B98] Wu Y. San Vicente F. Huang K. Dhliwayo T. Costich D. E. Semagn K.. (2016). Molecular characterization of CIMMYT maize inbred lines with genotyping-by-sequencing SNPs. Theor. Appl. Genet. 129, 753–765. doi: 10.1007/s00122-016-2664-8 26849239 PMC4799255

[B99] Xu C. Ren Y. Jian Y. Guo Z. Zhang Y. Xie C.. (2017). Development of a maize 55 K SNP array with improved genome coverage for molecular breeding. Mol. Breed. 37. doi: 10.1007/s11032-017-0622-z PMC5311085 28255264

[B100] Yassue R. M. Carvalho H. F. Gevartosky R. Sabadin F. Souza P. H. Bonatelli M. L.. (2021a). On the genetic architecture in a public tropical maize panel of the symbiosis between corn and plant growth-promoting bacteria aiming to improve plant resilience. Mol. Breed. 41. doi: 10.1007/s11032-021-01257-6 PMC10236062 37309313

[B101] Yassue R. M. Sabadin F. Galli G. Alves F. C. Fritsche-Neto R. (2021b). CV-α: designing validations sets to increase the precision and enable multiple comparison tests in genomic prediction. Euphytica 217. doi: 10.1007/s10681-021-02831-x

[B102] Ye C. Y. Fan L. (2021). Orphan crops and their wild relatives in the genomic era. Mol. Plant 14, 27–39. doi: 10.1016/j.molp.2020.12.013 33346062

[B103] Zheng X. Levine D. Shen J. Gogarten S. M. Laurie C. Weir B. S. (2012). A high-performance computing toolset for relatedness and principal component analysis of SNP data. Bioinformatics 28, 3326–3328. doi: 10.1093/bioinformatics/bts606 23060615 PMC3519454

