# Supplementary material for: On the usefulness of mock genomes to define heterotic pools, testers, and hybrid predictions in orphan crops
Source: Front Plant Sci. 2023 Jun 2;14:1164555. doi: 10.3389/fpls.2023.1164555 (PMC10272588; doi:10.3389/fpls.2023.1164555)
Supplement: Supplementary file 1 [file DataSheet_1.pdf]

Supplementary Material

1 Supplementary Tables

Table S1. Kendall's correlation in the clustering of datasets.

	GBS-B73	GBS-Mock
**SNP-array**	0.99**	0.96**
**GBS-B73**	-	0.97**

SNP-array: Affymetrix® Axiom Maize Genotyping array; GBS-B73: genotyping-by-sequencing with SNP calling using B73 as reference genome; GBS-Mock: genotyping-by-sequencing with SNP calling using the mock reference built with all parental lines.

** Empirical significance level from permutations.

Table S2 Variance components and genomic heritability of traits from SNP datasets.

		GY	PH	EH
σ^2_a	**SNP-array**	0.14	34.27	31.55
	**GBS-B73**	0.15	30.36	31.34
	**GBS-Mock**	0.14	35.82	32.49
σ^2_d	**SNP-array**	0.07	5.83	1.95
	**GBS-B73**	0.09	7.88	3.08
	**GBS-Mock**	0.08	3.62	1.01
σ^2_r	**SNP-array**	0.17	17.92	6.70
	**GBS-B73**	0.16	18.02	7.03
	**GBS-Mock**	0.16	20.55	7.61
H^2	**SNP-array**	0.56	0.69	0.83
	**GBS-B73**	0.60	0.68	0.83
	**GBS-Mock**	0.57	0.66	0.81
h^2	**SNP-array**	0.36	0.59	0.78
	**GBS-B73**	0.37	0.54	0.76
	**GBS-Mock**	0.37	0.60	0.79

SNP-array: Affymetrix® Axiom Maize Genotyping array; GBS-B73: genotyping-by-sequencing with SNP calling using B73 as reference genome; GBS-Mock: genotyping-by-sequencing with SNP calling using the mock reference built with all parental lines.

GY: grain yield; PH: plant height: EH: ear.

$\hat{\sigma}_a^2$, $\hat{\sigma}_d^2$ and $\hat{\sigma}_r^2$: Additive, dominance and residual variances, respectively: H^2: broad-sense heritability; h^2: narrow-sense heritability.

Table S3 Coincidence in the division of the parental lines into heterotic groups among datasets.

		**GBS-B73**	**GBS-Mock**
**GY**	**SNP-array**	0.94**	1.00**
	**GBS-B73**	-	0.94**
**PH**	**SNP-array**	0.94**	0.93**
	**GBS-B73**	-	0.87**
**EH**	**SNP-array**	0.94**	0.93**
	**GBS-B73**	-	0.87**

SNP-array: Affymetrix® Axiom Maize Genotyping array; GBS-B73: genotyping-by-sequencing with SNP calling using B73 as reference genome; GBS-Mock: genotyping-by-sequencing with SNP calling using the mock reference built with all parental lines.

** Significant at the 0.01 probability level by the t-test.

Table S4 SCA's and GCA's correlation of the lines between the SNP datasets.

		GBS-B73	GBS-Mock
		SCA	
GY	SNP-array	0.97**	0.96**
	GBS-B73	-	0.97**
PH	SNP-array	0.97**	0.96**
	GBS-B73	-	0.97**
EH	SNP-array	0.97**	0.96**
	GBS-B73	-	0.97**
		GCA	
GY	SNP-array	0.99**	1.00**
	GBS-B73	-	0.99**
PH	SNP-array	1.00**	1.00**
	GBS-B73	-	1.00**
EH	SNP-array	1.00**	1.00**
	GBS-B73	-	1.00**

SNP-array: Affymetrix® Axiom Maize Genotyping array; GBS-B73: genotyping-by-sequencing with SNP calling using B73 as reference genome; GBS-Mock: genotyping-by-sequencing with SNP calling using the mock reference built with all parental lines.

** Significant at the 0.01 probability level by the t-test.

2 Supplementary Figures

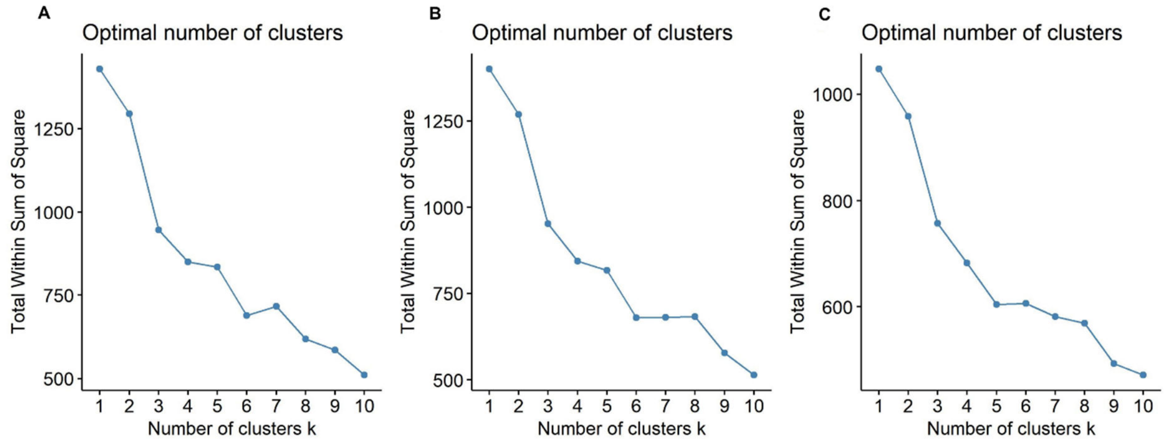

Supplementary Figure 1. Optimal number of clusters formed among the 330 parental lines for all SNP datasets by Total Within Sum of Square (WSS) method. **A** SNP-array; **B** GBS-B73; **C** GBS-Mock.

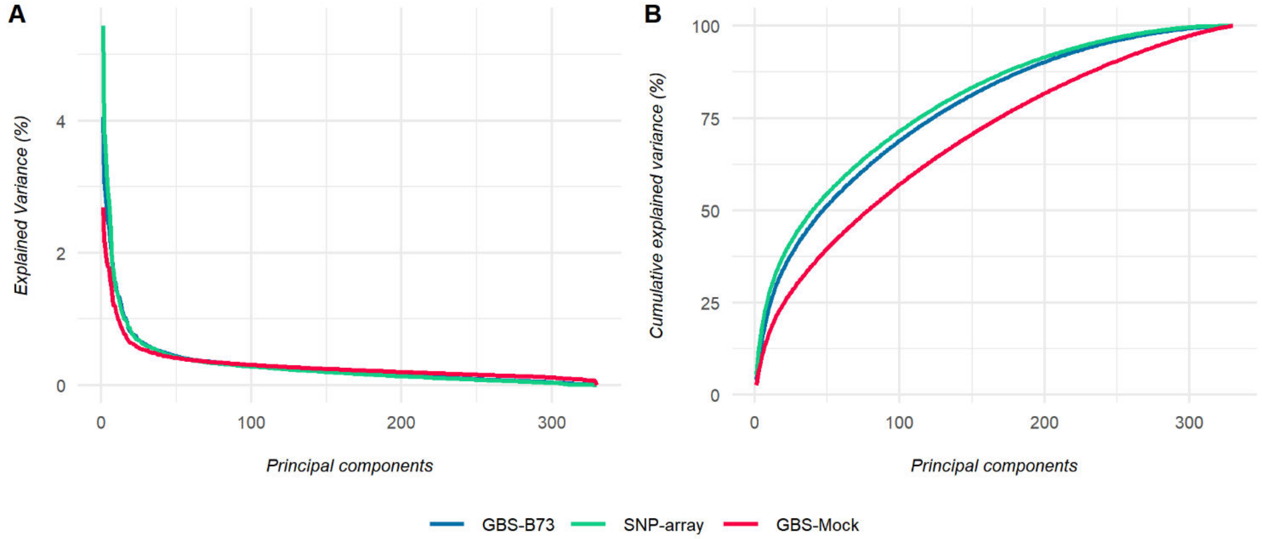

Supplementary Figure 2. A Variance explained by the principal components (PCA) from SNP-array, GBS-B73 e GBS-Mock SNP datasets for 330 tropical parental lines; **B** Cumulative explained variance estimated by principal components from SNP-array, GBS-B73 e GBS-Mock SNP datasets for 360 tropical parental lines.

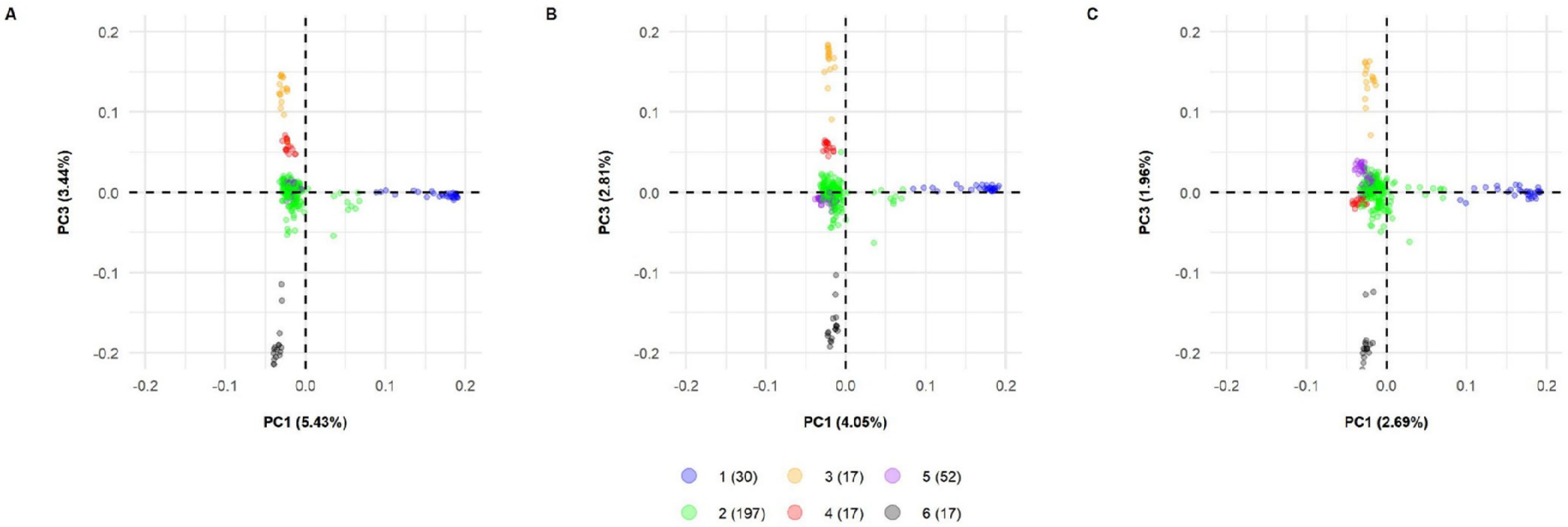

Supplementary Figure 3. Bi-plot the first and third principal components using all datasets for 330 tropical parental lines **A** SNP-array; **B** GBS-B73 and **C** GBS-Mock. Explained variance percentages of each principal component are in parentheses. Clusters were used to color-coded parental lines.

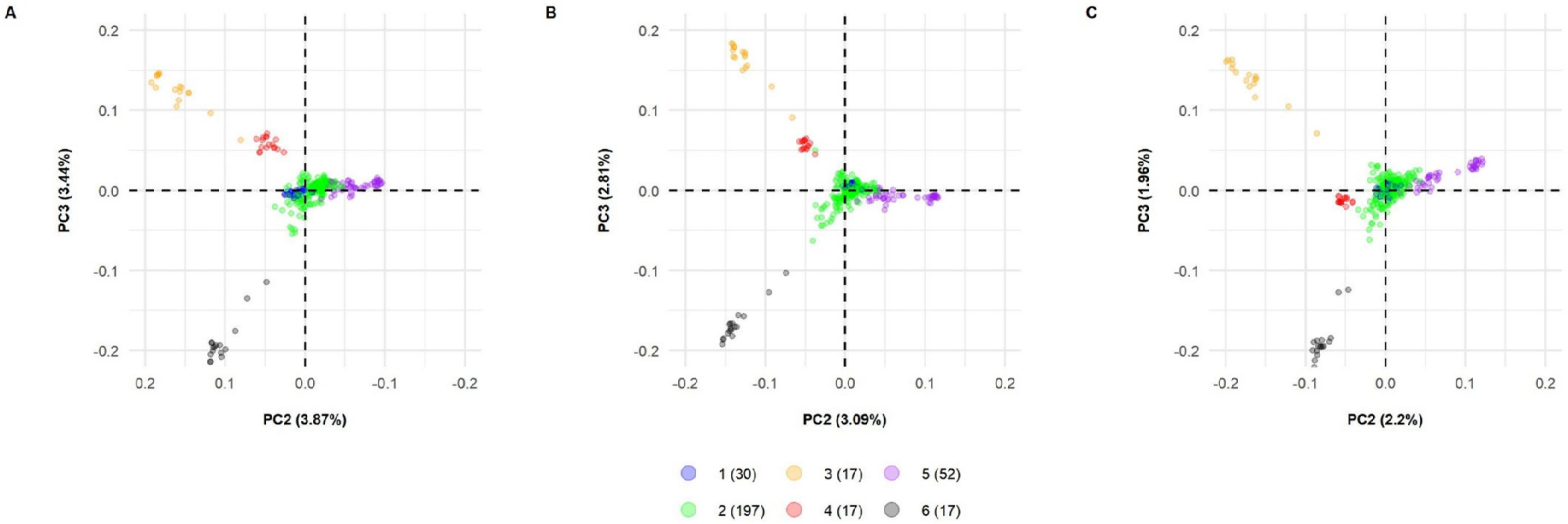

Supplementary Figure 4. Bi-plot the second and third principal components using all datasets for 330 tropical parental lines **A** SNP-array; **B** GBS-B73 and **C** GBS-Mock. Explained variance percentages of each principal component are in parentheses. Clusters were used to color-coded parental lines.

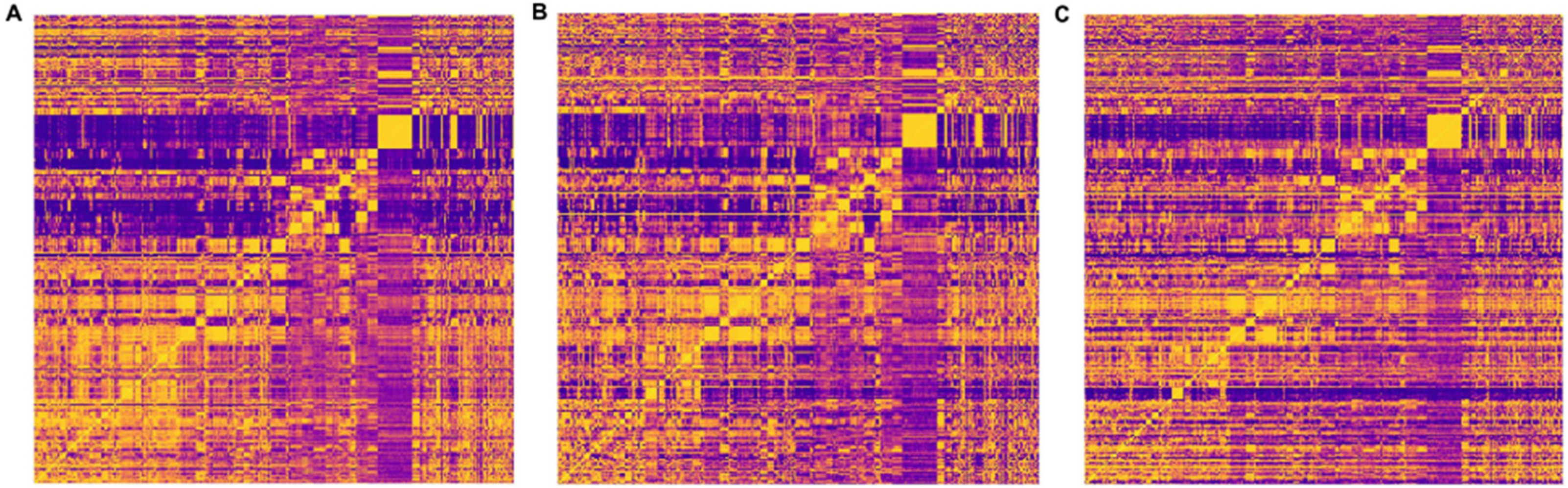

Supplementary Figure 5. Heatmaps of the Rogers genetic distance matrices estimated from **A** SNP-array, **B** GBS-B73 and **C** GBS-Mock-All SNP datasets for 330 tropical parental lines. Lines and columns of each plot were clustered according to the Euclidian distance performed in the Roger genetic distance matrix from the SNP-array dataset.

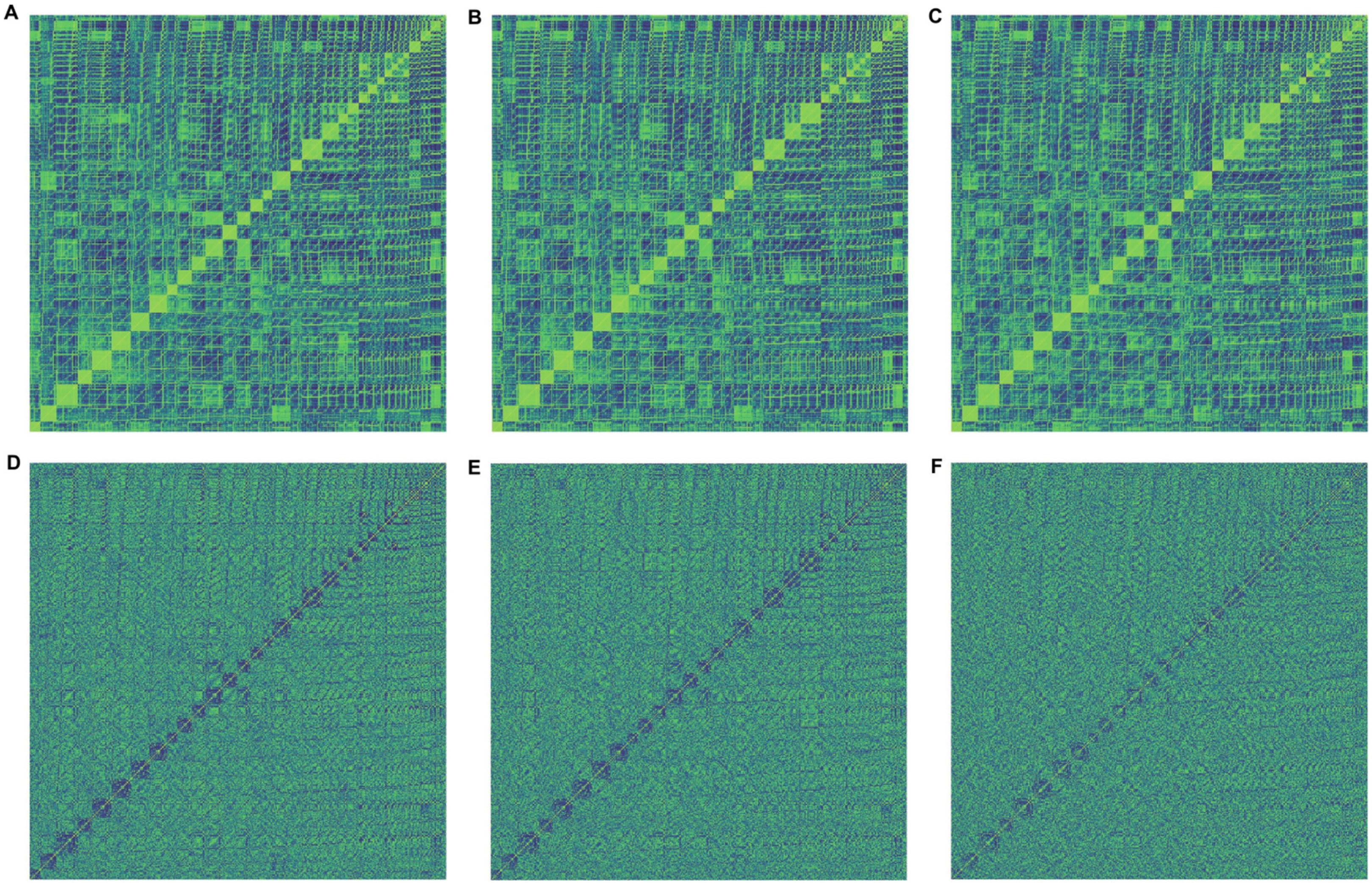

Supplementary Figure 6. Heatmaps of the (**A**, **B** and **C**) additive genomic relationship ($\boldsymbol{Ga}$), and (**D**, **E** and **F**) dominance genomic relationship ($\boldsymbol{Gd}$) matrices estimated from (**A** and **D**) SNP-array, (**B** and **E**) GBS-B73, and (**C** and **F**) GBS-Mock SNP datasets for 751 tropical maize single crosses. Lines and columns of each plot were clustered according to the Euclidian distance performed in the genomic relationship matrices from the SNP-array dataset.
